# Habitat-specificity in SAR11 is associated with a few genes under high selection

**DOI:** 10.1093/ismejo/wraf216

**Published:** 2025-10-11

**Authors:** Sarah J Tucker, Kelle C Freel, A Murat Eren, Michael S Rappé

**Affiliations:** Hawai‘i Institute of Marine Biology, School of Ocean and Earth Science and Technology, University of Hawai‘i at Mānoa, Kāne‘ohe, HI 96744, United States; Marine Biology Graduate Program, University of Hawai‘i at Mānoa, Honolulu, HI 96822, United States; Josephine Bay Paul Center for Comparative Molecular Biology and Evolution, Marine Biological Laboratory, Woods Hole, MA 02543, United States; Helmholtz Institute for Functional Marine Biodiversity, 26129 Oldenburg, Lower Saxony, Germany; Alfred Wegener Institute, Helmholtz Centre for Polar and Marine Research, 27570 Bremerhaven, Lower Saxony, Germany; Hawai‘i Institute of Marine Biology, School of Ocean and Earth Science and Technology, University of Hawai‘i at Mānoa, Kāne‘ohe, HI 96744, United States; Josephine Bay Paul Center for Comparative Molecular Biology and Evolution, Marine Biological Laboratory, Woods Hole, MA 02543, United States; Helmholtz Institute for Functional Marine Biodiversity, 26129 Oldenburg, Lower Saxony, Germany; Alfred Wegener Institute, Helmholtz Centre for Polar and Marine Research, 27570 Bremerhaven, Lower Saxony, Germany; Institute for Chemistry and Biology of the Marine Environment, University of Oldenburg, 26129 Oldenburg, Lower Saxony, Germany; Marine ‘Omics Bridging Group, Max Planck Institute for Marine Microbiology, 28359 Bremen, Germany; Hawai‘i Institute of Marine Biology, School of Ocean and Earth Science and Technology, University of Hawai‘i at Mānoa, Kāne‘ohe, HI 96744, United States

**Keywords:** SAR11, niche-partitioning, environmental selection, microbial metabolism, pangenomics, marine bacteria, *Pelagibacter*

## Abstract

The order *Pelagibacterales* (SAR11) is the most abundant group of heterotrophic bacteria in the global surface ocean, where individual sublineages likely play distinct roles in oceanic biogeochemical cycles. Yet, understanding the determinants of niche-partitioning within SAR11 has been a formidable challenge due to the high genetic diversity within individual SAR11 sublineages and the limited availability of high-quality genomes from both cultivation and metagenomic reconstruction. Through an integrated metapangenomic analysis of 71 new SAR11 isolate genomes and a time-series of metagenomes from the prominent source of isolation, we reveal an ecological and phylogenetic partitioning of metabolic traits across SAR11 genera. We resolve distinct habitat-preferences among genera for coastal or offshore environments of the tropical Pacific and identify a handful of genes involved in carbon and nitrogen metabolisms that appear to contribute to these contrasting lifestyles. Furthermore, we find that some habitat-specific genes experience high selective pressures, indicating that they are critical determinants of SAR11 fitness and niche differentiation. Together, these insights reveal the underlying evolutionary processes shaping niche-partitioning within sympatric and parapatric populations of SAR11 and demonstrate that the immense genomic diversity of SAR11 bacteria naturally segregates into ecologically and genetically cohesive units, or ecotypes, that vary in spatial distributions in the tropical Pacific.

## Introduction

Bacterial populations typically harbor extensive variation in gene content that can be difficult to delineate into discrete ecological or evolutionary units, despite the significant impact such differences may have on the biogeochemical roles, ecological interactions, and fitness of these microorganisms [1–3]. Comparative genomics has enabled the identification of gene content variation that underpins distinct ecologies and contributes to the process of niche diversification [4–6]. However, these analyses most often use a collection of genomes isolated from diverse environments where genes have evolved under a range of selective pressures [7], which can limit the capacity to associate variations in gene content with specific forces of selection and the identification of crucial genes that likely serve as determinants of fitness.

The bacterial order *Pelagibacterales* (SAR11) is one of the most abundant and diverse bacterial groups in the surface oceans [8–10], where even a single SAR11 population can exhibit substantial sequence diversity in the environment [11]. Defining the genomic variation underlying distinct spatial distributions of SAR11 populations and how gene content differs across environmental conditions is vital to further understanding ocean biogeochemical cycling, eco-evolutionary relationships, and the biology of a ubiquitous and dominant lineage in the surface oceans. This task remains difficult because assessing the loss or gain of ecologically relevant genes or metabolic pathways in SAR11 necessitates a robust collection of high-quality genome sequences that represent a spectrum of both ecological and genomic diversity. SAR11 cells are historically recalcitrant to laboratory cultivation, and as such only a small portion of the total diversity of naturally occurring SAR11 populations can be interrogated via high-quality genomes such as those from isolated strains [12]. Yet even with a small set of isolate genomes, SAR11 has become a key model to help elucidate fundamental processes of ecology and evolution such as genome streamlining [13], marine oligotrophy [14], coevolution [15, 16], drivers of genetic diversity and evolution within organisms of large population sizes [11, 17], protein structure-aware investigations of microbial population genetics [18], and ocean biogeochemical cycling [19–21].

In our recent work [22], we filled the gap in the available resources by sequencing the genomes of new SAR11 isolates that originated from a geographically constrained environment within the tropical Pacific, and developed a phylogenomic backbone that unites evolution with ecology to understand this diverse clade. This led to the delineation of four families within SAR11, including the family *Pelagibacteraceae*, which encompasses the historical SAR11 subgroups Ia and Ib [22]. Here, we illuminate metabolic diversity found within *Pelagibacteraceae* and identify the determinants of niche differentiation within co-occurring *Pelagibacteraceae* populations by combining 71 of the new isolates with 21 previously sequenced *Pelagibacteraceae* isolate genomes. By taking advantage of metagenomes from the prominent source of isolation, we apply an integrated ‘omics analysis to unveil differences in the metabolic features of *Pelagibacteraceae* across coastal and offshore habitats, and identify metabolic genes under high selective pressures.

## Materials and methods

### Pangenome analyses

Publicly available *Pelagibacteraceae* isolate genomes downloaded from the National Center for Biotechnology Information (NCBI) or the Joint Genome Institute were combined with genomes sequenced from newly isolated strains [22] (Supplementary File 1A). Genome completion and contamination were examined with CheckM v1.1.2 [23] and only isolates above 90% completion with less than 5% contamination [24] and fewer than 50 contigs were kept (Supplementary File 1A). High-quality *Pelagibacteraceae* genomes were used to construct a pangenome in anvi’o v8.0 [25] following previously described pipelines [26]. Briefly, an anvi’o database was created using anvi-gen-contigs-db and Prodigal v2.6.3 [27] was used to identify open reading frames (ORFs) from contigs. Single-copy core genes were identified using HMMER v3.2.1 [28]. ORFs with associated functions were annotated from NCBI’s Clusters of Orthologous Groups (COGs) [29] and a customized HMM database of Kyoto Encyclopedia of Genes and Genomes (KEGG) orthologs (KOfams) [30, 31]. The pangenome was created with anvi-pan-genome, which uses NCBI’s Basic Local Alignment Search Tool [32] to quantify the similarity between pairs of gene clusters and the Markov Cluster algorithm (MCL) [33] to define homologous gene clusters with a MCL inflation parameter of 2. The pangenomes were visualized using the command anvi-display-pan and summary tables exported using the command anvi-summarize.

Genome-wide average nucleotide identity (gANI) was estimated using FastANI in anvi’o [34]. A phylogenomic tree of the *Pelagibacteraceae* was estimated using IQ-Tree v2.12 [35] using 1000 ultrafast bootstraps and model LG + F + R10 from a concatenated alignment of a custom gene set for SAR11 (SAR11_165) [22]. Phylogenies were rooted and branches trimmed in R v 4.4.1 [36] using treeio v1.28.0 [37] and visualized in R using phytools v2.3.0 [38] to examine the phylogenomic relationships between *Pelagibacteraceae* groups detected in our environmental study.

Gene clusters found in 100% of *Pelagibacteraceae* genomes were considered core, whereas gene clusters found in only one genome were considered singletons. Gene clusters were also assessed as genus-specific (shared among all representatives of a genus and not found in other genera) and multi-genus-specific (shared among all representatives of two or more genera and not found among all others). Shared gene content between genera was assessed using ComplexUpset v 1.3.3 [39].

### Metagenomic read recruitment and environmental analyses

**Figure 1:**
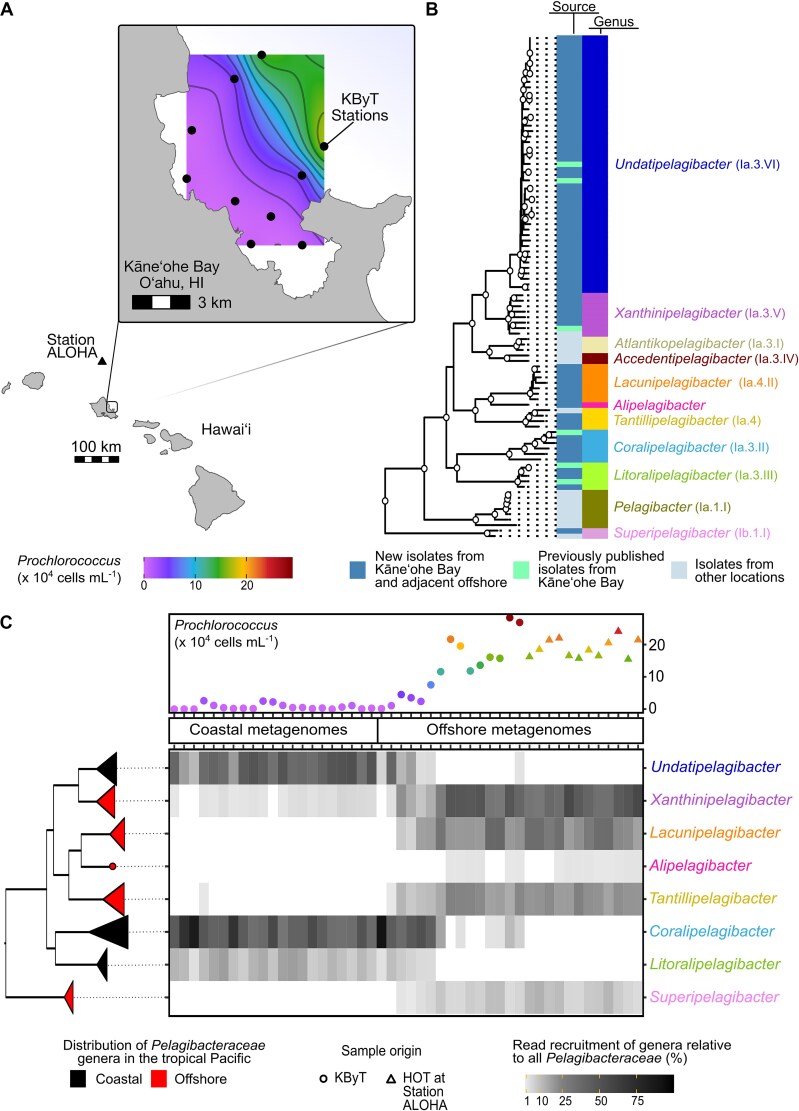
*Pelagibacteraceae* show polyphyletic habitat-preferences for coastal or offshore environments across KByT. (A) The location of the Kāneʻohe Bay Time-series (KByT) sampling stations and the Hawai‘i Ocean Time-series (HOT) at Station ALOHA. KByT spans a steep biogeochemical gradient, as reflected by the dramatic increase in *Prochlorococcus* cellular abundances immediately offshore. (B) Eleven genera are represented by genome-sequenced *Pelagibacteraceae* isolates, with the majority isolated from coastal Kāneʻohe Bay and the adjacent offshore. Nodes with circles represent ≥90% bootstrap support. Historically referenced clade names for *Pelagibacteraceae* genera are provided in parentheses. (C) The relative read recruitment of *Pelagibacteraceae* isolate genomes summed at the level of genera for metagenomes from KByT and Station ALOHA. Metagenomes grouped into two clusters by environmental parameters (coastal and offshore), coinciding with the gradient in *Prochlorococcus* cellular abundances. *Pelagibacteraceae* genera showed distinct distribution patterns in the coastal and offshore waters that are distributed polyphyletically. Read recruitment <1% not shown. The order of metagenomes presented follows the order in Supplementary file 1e.

To examine the distribution of *Pelagibacteraceae* genera within the environments from where the majority were isolated, metagenomic read recruitment was conducted using surface ocean metagenomes from 10 stations within and adjacent to Kāneʻohe Bay, Hawai‘i (Fig. 1) collected as part of the Kāneʻohe Bay Time-series (KByT; PRJNA971314) [40, 41]. In addition, metagenomes collected in the surrounding North Pacific Subtropical Gyre at Station ALOHA, a sampling location of the Hawaii Ocean Time-series (Fig. 1, Supplementary File 1C, PRJNA352737) [42] were also used. Metagenomes were competitively mapped with Bowtie2 v2.3.5 [43] to the anvi’o contig database of *Pelagibacteraceae* isolate genomes. The anvi-profile function stored coverage and detection statistics of each *Pelagibacteraceae* genome found in the KByT and Station ALOHA metagenomic samples and the anvi-meta-pan-genome function [26] was used to bring together the pangenomic information with the read recruitment data.

To evaluate the distribution of individual genomes, a detection metric—the proportion of the nucleotides in a given sequence that are covered by at least one short read—was used to define whether a population was detected in a metagenomic sample. A detection value of at least 0.25 was used as criteria to eliminate false positives that could arise if an isolate genome was falsely found within a sample [44].

The average depth of coverage excluding nucleotide positions with coverages in the 1st and 4th quartiles (mean coverage Q2Q3) was mapped for each genome in each sample. To avoid biased estimates of coverage that can occur due to highly recruiting accessory genes, read recruitment was analyzed using only single-copy genes core to each genus. Using a custom script in R, gene clusters that were found within all genomes of the genus (e.g. core), but found only in a single copy within each genome were identified. Then the mean coverage Q2Q3 read recruitment data per gene were then subsetted for only single-copy core genes. The mean coverage Q2Q3 of each single-copy core gene was summed per genome per sample and then this value was normalized per genome per sample, based on whether the genome was detected at >0.25 [44]. Next, when evaluating read recruitment at the genus-level, the read recruitment was summed for all genomes in a genus per sample. These outputs were then divided across the total *Pelagibacteraceae* read recruitment for the sample to yield a relative estimate of each genome or genus within a sample. For the genus with only a single genome representative (*Alipelagibacter*), the single-copy core genes shared with the HIMB1483 genome and nearest neighbor, genus *Lacunipelagibacter*, were used.

K-means clustering analysis of a scaled matrix of biogeochemical conditions was used to characterize the environmental background from which the metagenomic data were derived. Metadata from Station ALOHA were downloaded from https://hahana.soest.hawaii.edu/hot/hot-dogs/ (accessed 11 Oct 2021). All KByT (*n* = 158) and Station ALOHA (*n* = 34) surface seawater samples (depth of <30 m) with data for flow cytometrically determined cellular abundance, chlorophyll *a* concentrations, silicate concentrations, temperature, pH, and salinity were included in the analyses (Supplementary File 1C). The number of clusters were estimated using the kmeansruns command in the R fpc v2.2.13 package [45] using a Calinski Harabasz index. Principal component analysis was conducted using the prcomp function in R v4.4.1 [36] and visualized using ggbiplot in the ggplot2 v3.5.1 [46].

The sampling locations of the Kāneʻohe Bay Time-series and Station ALOHA were mapped using R with geom_sf from ggplot2, using geospatial data of the main Hawaiian Islands (USGS Digital Line Graphs). To visualize *Prochlorococcus* cellular abundance across the Kāneʻohe Bay Time-series, mba.surf from MBA v0.1.2 [47] was used to interpolate data over the KByT stations.

### Functional inferences from *Pelagibacteraceae* genera

Functional enrichment analysis was performed in anvi’o and has been described previously [48]. Briefly, the anvi-compute-functional-enrichment command, as well as the anvi-script-gen-function-matrix-across-genomes, was used to assess the enrichment of COG, KEGG orthologs (KOs), and KEGG modules across genomes and genus affiliations. The degree of completeness of individual KEGG modules [49, 50] in the genomes and genera was evaluated using anvi-estimate-metabolism [51], prior to anvi-compute-functional-enrichment. The functional pangenome was visualized using anvi-display-functions. Central and ancillary carbon metabolisms, amino acid, vitamin, and cofactor biosynthesis, and genes to cope with nutrient, organic carbon, osmotic, and oxidative stress were examined across the *Pelagibacteraceae* isolate genomes. The distribution of functional genes were evaluated for each genus as follows: core genes (found in all genomes of a genus), intermediate genes (found in 15–99% of genomes), and rare genes (found in less than 15%). Heatmaps of the metabolic gene distribution across genera were made in the R package pheatmap v1.0.12 [52].

### Quantification of environmental selective pressures within *Pelagibacteraceae* genes

To evaluate the selective pressures on individual metabolic genes of interest, the proportion of non-synonymous to synonymous (pN/pS) sites per gene across genomes was evaluated. The pipeline followed those previously developed [18]. Briefly, contig databases from the type genomes of each of the *Pelagibacteraceae* genera [22] were first functionally annotated in anvi’o (see above) and metagenomic reads from deeply sequenced metagenomes from either coastal Kāneʻohe Bay or the adjacent offshore were non-competitively recruited using Bowtie2 v2.3.5 [43] (Supplementary File 1C). For *Tantillipelagibacter* and *Supripelagibacter,* a taxonomic technicality did not allow for the first isolate genomes sequenced from these genera to be used as type material, but were nonetheless used here to represent these genera [22]. The flag --profile-SCVs in anvi-profile was included to recover the single codon variants from the environment. Next, the program anvi-gen-variability-profile was used to export the single codon variants for each variable position in each gene across every metagenome using the flags --engine CDN, --include-site-pnps, and --kiefl-mode [18]. Finally, the program anvi-get-pn-ps-ratio with --min-coverage 20 was used to recover pN/pS values for each gene that was covered at least 20× in each sample. In parallel, the program anvi-summarize with the --init-gene-coverages flag was used to acquire coverage statistics for all genes (whether they were variable or not). The program anvi-export-functions exported functional annotations. These steps enabled us to link environmental population genetic variants with gene coverage and function.

To evaluate whether a single genome representative contained similar pN/pS gene values as other genomes within a given genus, these steps were repeated for all representatives of the genus sharing the lowest minimum gANI (among the genera that were abundant in the tropical Pacific): genus *Coralipelagibacter*. The exported data files were brought into R, where variation in pN/pS per sample, per genome, and across genomes of the same genus was evaluated. Relationships between pN/pS and coverage were examined and genes per genome were ranked from lowest pN/pS value to highest and visualized in R using ggplot2 v3.5.1 [46]. Figures were made in R v4.4.1 [36] or anvi’o [25] and further edited with Inkscape (https://inkscape.org/).

## Results

### Ecology and evolution of *Pelagibacteraceae* reveals strong habitat-specificity

Our analysis of 92 high-quality genomes from strains isolated predominantly from coastal Kāneʻohe Bay, Oʻahu, Hawai‘i, and the adjacent offshore revealed that the genomic diversity of cultured *Pelagibacteraceae* partitions into 11 discrete monophyletic clusters, seven of which include isolates from the Kāneʻohe Bay Time-series (KByT; Fig. 1, Fig. S1, Supplementary File 1A). The 92 genomes belong to both previously described and new clades, which we recently described as 11 genera [22] (Fig. 1, Supplementary File 1A). The within-genus genome-wide average nucleotide identity (gANI) differed among genera, with the lowest within-genus average gANI found in *Accedentipelagibacter* (87.1 ± 0.0%, mean ± SD, *n* = 2) and the highest within-genus average gANI found in *Litoralipelagibacter* (95.8 ± 1.4, *n* = 5, Supplementary File 1B). The average of all within-genus genome-wide average nucleotide identities was 92.3 ± 2.7% (*n* = 10).

We investigated the biogeography of *Pelagibacteraceae* isolate genomes through read recruitment with metagenomes from KByT and Station ALOHA in the adjacent North Pacific Subtropical Gyre. This revealed that the seven genera within the *Pelagibacteraceae* that contained KByT isolate genomes were commonly detected within the KByT system, whereas the three genera not containing KByT isolates were rarely detected, if at all (Fig. S1). K-means cluster analysis of biogeochemical parameters from 192 KByT and Station ALOHA surface ocean samples, including 48 samples with corresponding metagenomes, delineated two clusters that we labeled as coastal and offshore because of their distinct biogeochemical characteristics and geographic origins (Fig. S2, Supplementary File 1C, Supplementary Note 1). Briefly, the coastal cluster was characterized by elevated chlorophyll *a*, silicate, phosphate, and nitrate+nitrate concentrations; increased cellular abundances of heterotrophic bacteria, photosynthetic picoeukaryotes, and *Synechococcus*; and depressed salinity, pH, and *Prochlorococcus* cellular abundances compared to the offshore cluster (Fig. S2, Supplementary File 1D).

Congruent with the grouping of biogeochemical characteristics, the patterns of genome distribution revealed a clear dichotomy where each genus was highly present in either the offshore or coastal environment, but not both (Fig. 1C, Supplementary File 1E). Genera with higher abundances in the coastal environment, hereafter referred to as “coastal genera”, appear in polyphyletic clades, suggesting that coastal genera evolved multiple times across the evolutionary history of the *Pelagibacteraceae* (Fig. 1C). The patterns of distribution were consistent for all genomes within the same genus (Fig. S3), and genomes that belonged to the same genus generally recruited similar proportions of reads from the environment. All genomes from the genus *Coralipelagibacter* were among the most abundant within the coastal environment (8.4 ± 6.8%, mean ± SD, *n* = 21, Fig. S3). However, the *Coralipelagibacter* genome HIMB1412 had a particularly high relative abundance in the coastal environment (19.9 ± 9.4%, mean ± SD, *n* = 21, Fig. S3), which was roughly 2.5–4.3 times more abundant than the average abundances of the other *Coralipelagibacter* genomes. Although we consistently detected all isolate genomes of *Xanthinipelagibacter* in the offshore environment, four of eight genomes (HIMB83, FZCC0015, HIMB1456, and HIMB2250) were also frequently detected in the coastal environment (Fig. S3). Regardless, their relative abundances were low in coastal samples (0.4 ± 0.5%, mean ± SD) and sharply increased offshore (3.5 ± 2.0%, mean ± SD; Fig. S3), further supporting the characterization of *Xanthinipelagibacter* as an offshore genus.

Overall, our metagenomic read recruitment reveals that *Pelagibacteraceae* communities partition between coastal Kāneʻohe Bay and adjacent offshore waters, that these distinct communities are driven by differences in the distribution of individual *Pelagibacteraceae* genera, and that coastal genera (*Undatipelagibacter*, *Coralipelagibacter*, and *Litoralipelagibacter*) are distributed polyphyletically across the *Pelagibacteraceae*. Recent studies have also detailed the dominance of *Xanthinipelagibacter*, *Lacunipelagibacter*, and *Tantillipelagibacter* in low-latitude, open ocean waters [11, 12, 22] and support the classification of *Coralipelagibacter* and *Litoralipelagibacter* as coastal specialists in regions beyond KByT [22].

### *Pelagibacteraceae* pangenome includes differentially enriched genes and functions within coastal and offshore genera

**Figure 2:**
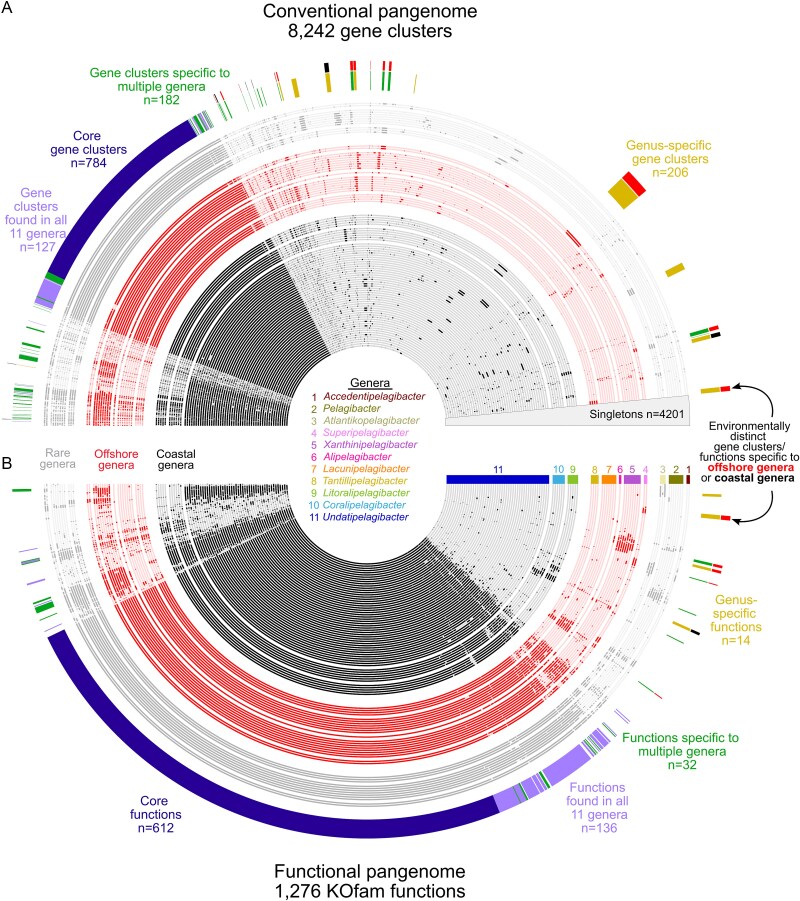
*Pelagibacteraceae* genomes share high overlap in genes and functions among ecologically distinct groups. The presence and absence of gene clusters and KOfams are shown across (A) a conventional pangenome and (B) a functional pangenome, respectively. Genomes (inner rings) are grouped by genus and further grouped by ecological distribution in the tropical Pacific as coastal, offshore, or rare. The distribution of the gene pool and functions in the second most outer layer (e.g. core, genus-specific) show that the gene pool and functions are shared extensively across all 11 genera and 92 genomes. The outer layer shows the sparse environmentally distinct portions of the gene pool and functional pool that are specific to coastal or offshore genera.

To investigate the potential determinants of fitness that may explain the distinct ecological patterns of distribution, we sought to characterize the genomic diversity and functional gene content of *Pelagibacteraceae* isolates. We utilized a pangenomic approach that partitioned all genes across all *Pelagibacteraceae* genomes into *de novo* gene families, or “gene clusters”, based on amino acid sequence homology. This analysis resulted in 8242 gene clusters across all 92 genomes, of which 784 were core among all *Pelagibacteraceae* and made up the majority (52%–63%) of gene content found in any individual genome (Fig. 2A; Supplementary File 2A). Half of the pangenome was composed of gene clusters that were singletons (4201 of 8242; Fig. 2A). Between the extremes of gene clusters that occur in every genome and those that occur only in one, we found 206 accessory gene clusters that were “genus-specific” (21 ± 27, mean ± SD per genus; Fig. 2A; Supplementary File 2B), as they were present among all representatives of a single genus, but absent from genomes belonging to any other genus. The 206 genus-specific gene clusters were not distributed uniformly across genera, as 95 were unique to the genus *Superipelagibacter* alone (Supplementary File 2B). However, this number is likely an overestimation of the true *Superipelagibacter* genus-specific traits since this particular genus was represented by only two genomes, and the number of genus-specific gene clusters negatively correlated with the number of genomes in a genus (*R*^2^adj = 0.37, *F* value = 6.2, *P* value = .037; Pearson’s correlation). Gene clusters that were core to multiple genera (e.g. present in every genome for two or more genera, but absent in every other) were also not a dominant feature in the pangenome (Fig. 2A). They only represented 182 gene clusters, and were generally uniformly distributed across many different groupings of genera (Fig. 2A; Supplementary File 2B). Focusing on taxonomically constrained gene clusters (e.g. genus-specific gene clusters and gene clusters specific to multiple genera), we could further assign gene clusters to coastal or offshore categories based on the environmental preference of the genomes in which they occurred, finding 38 and 176 gene clusters that were specific to coastal genera and offshore genera, respectively (Fig. 2A, Supplementary File 2B).

We focused on genes with well-characterized roles in known metabolisms by examining those that share orthology with genes described in KEGG [30] through KOfam models [31] and generating a “functional pangenome” (Fig. 2B). The *Pelagibacteraceae* functional pangenome grouped genes based on functional identity rather than amino acid sequence similarity and consisted of 1276 KOfam functions, with a large core (612 KOfams; Fig. 2B). A subset of the remaining non-core functions were still shared widely across all 11 genera with a relatively uniform distribution (*n* = 136; Fig. 2B) or were singletons (*n* = 70). Similar to the conventional pangenome, the functional pangenome contained very few genus-specific functions (14 total among all genera; 0–5 KOfams per genus; Fig. 2B) and a limited number of functions that were core to multiple genera (e.g. present in every genome for two or more genera, but absent in every other; 32 KOfams total; Fig. 2B; Supplementary File 2C). Only three KOfam functions were specific to coastal genera and 13 KOfam functions were specific to offshore genera (Fig. 2A; Supplementary File 2C).

Pangenomic analysis using conventional and functional approaches revealed a large overlap in gene content among ecologically distinct genera, with subtle but consistent genus-specific features. The high overlap in gene content across the *Pelagibacteraceae* and small number genus-specific gene clusters suggests differentiation between genera of the *Pelagibacteraceae* may be determined by only a small portion of the genome. The polyphyletic distribution of habitat-preference (Fig. 1C) supports that genes and functions that are linked to success in these different habitats likely evolved multiple times through independent processes. Given this evolutionary trajectory, it is highly plausible that instead of vertical inheritance, horizontal gene transfer led to the acquisition of most of these habitat-specific genes and subsequent high rates of recombination and strong selective forces resulted in the increased prevalence among *Pelagibacteraceae* in a given ecological niche. This process, referred to as gene-specific sweeps, frequently results in a more subtle display of habitat-specific traits [53], as is observed here. To further examine the potential importance of these environmentally distinct functions in providing advantages to *Pelagibacteraceae* prevalent in coastal or offshore habitats of the tropical Pacific, we next focused on the subset of the environmentally distinct functions characterized here that are involved in nutrient and organic carbon utilization and cellular stress. Furthermore, in examining the functions involved in nutrient and organic carbon utilization and cellular stress, we broadened our investigation beyond the functions that were strictly found in coastal genera or strictly found in offshore genera (e.g. environmentally distinct functions), to also include functions that were enriched in either coastal or offshore genera.

### Potential determinants of habitat-specificity in *Pelagibacteraceae* include molybdenum utilization and nitrogen and carbon metabolism

Despite a high rate of water exchange, coastal Kāneʻohe Bay, and the adjacent offshore vary in nutrient availability, osmotic conditions (e.g. salinity, temperature, pH), and phytoplankton communities that determine the organic carbon pool [40, 54], suggesting that this would enforce distinct environmental pressures on coastal and offshore *Pelagibacteraceae* genera. To characterize ecologically relevant genes that may support coastal and offshore habitat-preferences of *Pelagibacteraceae* across the KByT system, we used an enrichment analysis to examine metabolic traits related to nutrient and organic carbon acquisition and cellular stress that were differentially distributed across genomes from either coastal or offshore genera. Genes that were core across genera of the *Pelagibacteraceae* or did not show easily distinguishable distributions (e.g. driven by phylogeny or environment) are further discussed in Supplementary Note 2. Unless noted otherwise, the following metabolic traits were found outside of hyper-variable regions of genomes and thus represent genes that are encoded within the relatively stable genomic regions of a given genus or set of genera.

#### Molybdenum enzyme utilization

**Figure 3:**
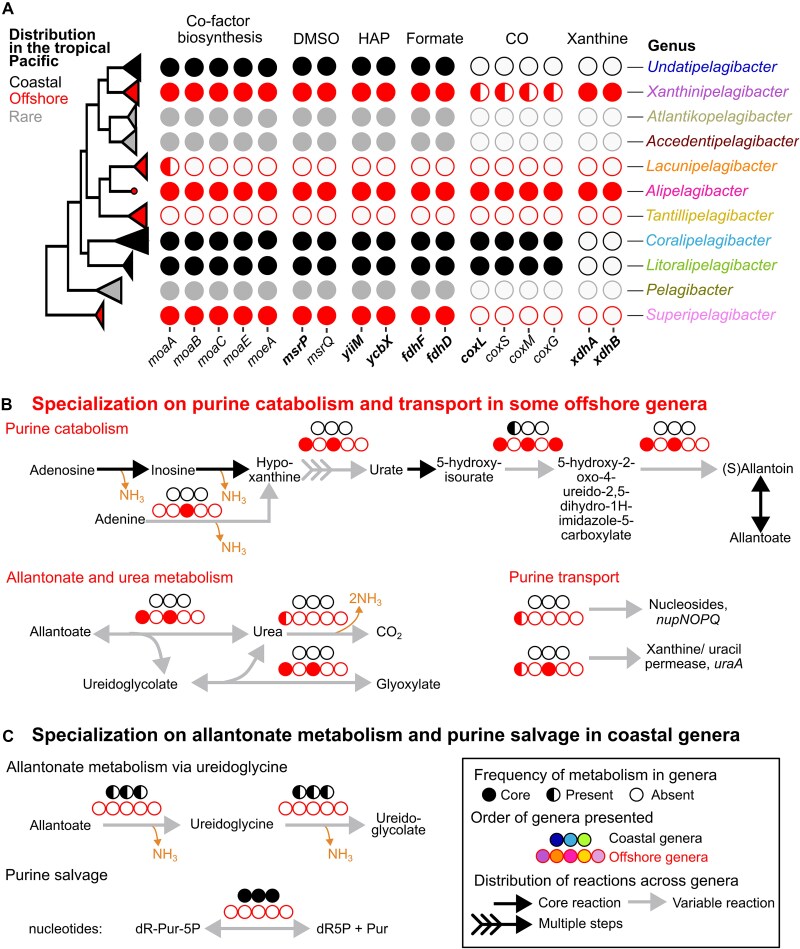
*Pelagibacteraceae* genera fill distinct niches in molybdenum-associated enzyme utilization. (A) The distribution of molybdenum cofactor (MoCo) biosynthesis genes and genes involved in metabolisms that require a molybdenum cofactor across *Pelagibacteraceae* genera. Metabolisms within the *Pelagibacteraceae* that require a molybdenum cofactor include those involved in the repair of methionine sulfoxides and the catalytic subunit of periplasmic dimethylsulfoxide (DMSO; *msrPQ*), the detoxification of 6-N-hydroxylaminopurine (HAP) to adenine (*yiiM*, *ycbX*), the oxidation of formate to carbon dioxide (*fdhFD*), the oxidation of carbon monoxide (*coxSMLG*), and the oxidation of purines via xanthine dehydrogenases (*xdhAB*). Genes predicted to encode proteins with molybdenum cofactor binding sites are noted in bold. (B) Genes encoding for purine catabolism and transport are only present in some offshore genera. (C) Genes encoding for allantonate metabolism and purine salvage are present only in coastal genera. MoCo: Molybdenum cofactor, DMSO: Dimethylsulfoxide, HAP: Hydroxylaminopurine, CO: Carbon monoxide, NH_3_: Ammonia, dR-Pur-5P: Purine 2′-deoxyribonucleoside 5′-monophosphate, Pur: Purine base, dRib5P: 2′-deoxyribonucleoside 5′-monophosphate, (D) R-Pur-1P: Beta-(deoxy)ribonucleosides.

Molybdenum-dependent enzymes are nearly ubiquitous among organisms from all domains of life, catalyze important oxidation–reduction reactions involved in carbon, sulfur, and nitrogen metabolisms, and require a cofactor scaffold to hold the molybdenum in place [55]. Although most *Pelagibacteraceae* genomes contained genes involved in molybdenum cofactor biosynthesis as well as molybdenum-dependent enzymes (Fig. 3A, Supplementary File 2D), these genes were missing from two offshore genera, *Lacunipelagibacter* and *Tantillipelagibacter*. In contrast to the lack of molybdenum-dependent enzymes found in the offshore *Lacunipelagibacter* and *Tantillipelagibacter*, other offshore genera, *Xanthinipelagibacter* and *Alipelagibacter*, appeared to increase reliance on molybdenum-dependent enzymes by uniquely harboring genes encoding molybdenum-dependent enzymes associated with the oxidation of purines (xanthine; *xdhAB*) (Fig. 3A, Supplementary File 2D, Supplementary Note 3). The distribution of molybdenum-dependent enzymes is polyphyletically distributed among offshore genera, where some offshore groups possess no molybdenum-dependent enzymes and others harbor all the molybdenum-dependent enzymes detected within *Pelagibacteraceae* (Fig. 3A). We hypothesize that the absence of molybdenum-dependent enzymes within the offshore *Lacunipelagibacter* and *Tantillipelagibacter*, which have some of the smallest genomes within *Pelagibacteraceae* (~1.2Mbp; Supplementary File 1A), is likely driven by streamlining selection to minimize metabolic requirements and genome size [13, 56]. Although molybdenum concentrations are not known to differ between waters of coastal Hawai‘i and the open ocean, the concentration of iron is reported to be at least one order of magnitude higher in waters of coastal Hawai‘i compared to the open ocean [57]. Because molybdenum cofactor biosynthesis and several molybdenum-dependent enzymes require iron–sulfur clusters [58], the distinct distribution of molybdenum-dependent enzymes between coastal and offshore *Pelagibacteraceae* may also be related to variable concentrations of iron in coastal and open ocean waters of the tropical Pacific.

#### Nitrogen metabolism

Purines are abundant in aquatic environments [59], contain a high number of nitrogen atoms per molecule [60], and likely contribute to niche-differentiation between coastal and offshore SAR11 [61]. Our metabolic reconstructions show that offshore genera *Xanthinipelagibacter* and *Alipelagibacter* harbored genes to catabolize purines through the utilization of a molybdenum-dependent xanthine dehydrogenase (Fig. 3B, Supplementary File 2E), as well as genes with functions predicted to support the efficient transport of nucleotides (*Xanthinipelagibacter*: *nupNOPQ* system; *Xanthinipelagibacter* and *Alipelagibacter*: xanthine/uracil permease *uraA*). To make hypoxanthine available for degradation, *Alipelagibacter* can convert adenine to hypoxanthine using adenine deaminase (*ade*) and both genera *Alipelagibacter* and *Xanthinipelagibacter* also uniquely possess a tRNA(adenine34) deaminase (*tadA*), which can convert adenine to hypoxanthine (Fig. 3B). Most *Xanthinipelagibacter* genomes (6 of 8) also harbor a urease system (*ureABCDEFG*) to liberate ammonia in the final steps of the xanthine degradation pathway.

In contrast to the purine degradation pathways found within the offshore genera, we found coastal genera specialize on the degradation of allatonate, a by-product of purine metabolism, as well as the recycling of nucleotides. Members of the coastal genus *Undatipelagibacter* (and a single *Coralipelagibacter* genome, HIMB5) have the functional capacity to degrade allantoate to (S)-ureiodoglycine and ammonia via an allantoate deiminase and ureidoglycine to ureidoglycolate and ammonia, via a nucleophile (Ntn)-hydrolase (Fig. 3C, Supplementary File 2E). The allantoin degradation genes found in coastal genomes characterized here appear to provide a source of nitrogen, and differ from those found in the offshore genera. All coastal genera possess a deoxynucleoside 5-monophosphate N-glycosidase (*rcl*) that putatively breaks the N-glycosidic bond of purine nucleotides (i.e. purine 2′-deoxyribonucleoside 5′-monophosphates; Fig. 3C). This differs from the purine nucleoside salvage pathway core to both coastal and offshore genera, which requires orthophosphate to cleave the N-glycosidic bond of beta-(deoxy)ribonucleosides to yield alpha-(deoxy)ribose 1-phosphate (Supplementary File 2E). All *Pelagibacteraceae* are missing genes to utilize deoxyribose-5-phosphate or deoxyribose-1-phosphate via a deoxyribose-phosphate aldolase (*deoC*). However, ribose-1-phosphate could be catabolized to D-fructose-6P and D-glyceraldehyde-3P (Supplementary File 2E).

**Figure 4:**
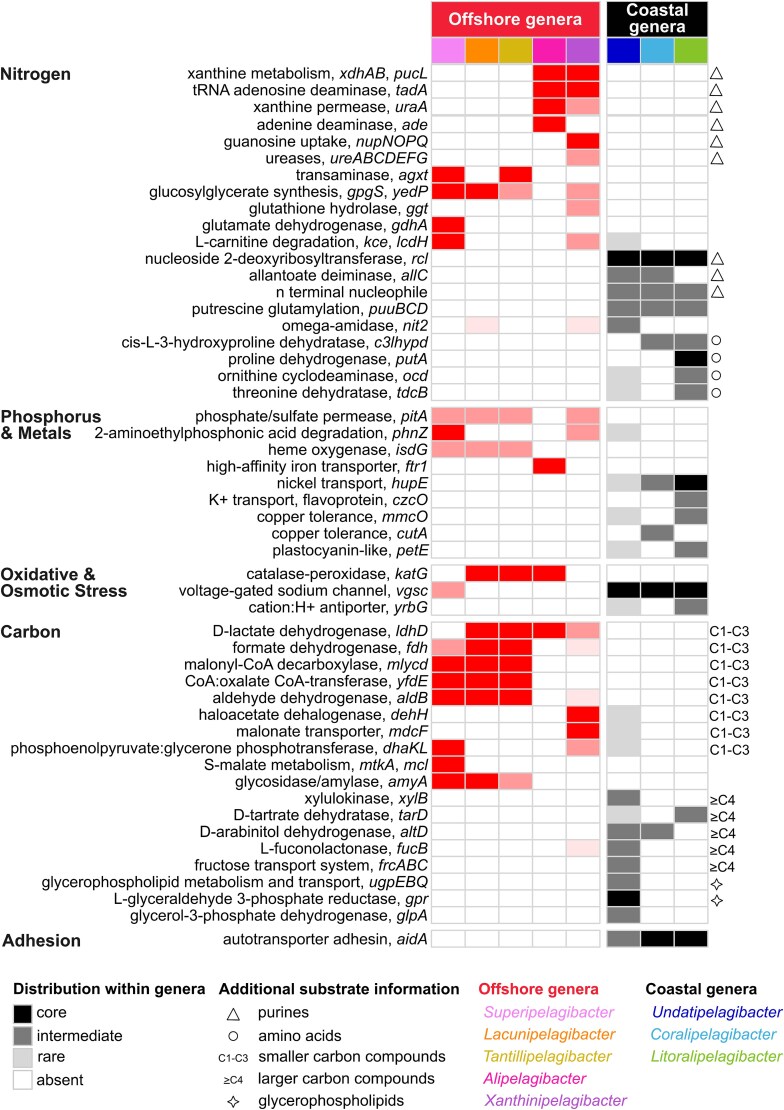
Metabolic determinants of habitat-specificity in *Pelagibacteraceae*. The distribution of metabolic genes associated with the acquisition of carbon, nitrogen, phosphorus, and metals and oxidative and osmotic cellular stress vary among coastal and offshore *Pelagibacteraceae* genera. Each column represents a genus, organized by genera that have offshore (first five columns) and coastal (last three columns) biogeographical distributions. Each row represents a metabolic gene(s) organized by their role in carbon, nitrogen, phosphorus, or metal acquisition or oxidative and osmotic cellular stress. The intensity of color indicates the prevalence of the metabolic gene(s) in each genus. Gene distribution patterns presented here are summarized in Supplementary File 2g.

In addition to differences in purine metabolism, offshore genera uniquely harbor a transaminase (*agxt*) that may help these cells to overcome auxotrophies for the biosynthesis of the amino acids glycine and serine (Fig. 4, Supplementary File 2F), which are common features across *Pelagibacteraceae*, but likely restrict important biosynthetic pathways including protein synthesis and central carbon metabolism [62]. Offshore genera also uniquely contain genes involved in glutamate deamination, glutathione and carnitine metabolism, and the synthesis of glucosylglycerate, which is used as an alternative osmolyte under nitrogen-limited conditions in cyanobacteria [63]. In contrast, the coastal genera contain nitrogen metabolisms generally involved in the deamination of various amino acids (e.g. hydroxyprolines, proline, ornithine, and threonine). A subset of coastal *Undatipelagibacter* genomes also have the potential to metabolize the polyamines putrescine and spermidine via *puuBCD* (Fig. 4). The *puuD* gene was located in hyper-variable regions within coastal *Undatipelagibacter* genomes.

#### Phosphate and metals

Some offshore *Pelagibacteraceae* genera exhibit a reduced genomic capacity to transport phosphate relative to coastal genera. High-affinity phosphate acquisition genes (*pstSCAB-phoU*; *phoB*; *phoR*) were core among coastal genera but only found within 55% of all offshore genomes. Offshore genomes lacking this system appear to rely solely on the *pitA* gene, a presumably lower-affinity proton-motive force permease of phosphate or sulfur (Fig. 4, Supplementary File 2F). Some offshore genera contain an increased genomic capacity to transport or capture iron relative to coastal genera. All *Pelagibacteraceae* contain an iron (III) transport system (*afuAB*), however the offshore *Alipelagibacter* genus alone possesses a high-affinity iron permease (*ftr1*) (Fig. 4, Supplementary File 2F). Offshore genera *Superipelagibacter*, *Lacunipelagibacter*, and *Tantillipelagibacter* also contain a heme oxidase, which could catalyze the oxidative degradation of the heme porphyrin ring to release iron. Unique genes to utilize copper, potassium, and nickel were found among the coastal genera (Fig. 4). We observed a copper-containing plastocyanin-like gene within a small subset of coastal genomes (Fig. 4; Supplementary File 2F). Plastocyanins serve as an electron carrier from cytochrome *f* to photosystem I and are generally found within eukaryotic phytoplankton and cyanobacteria [64]. We characterized homologs of the plastocyanin-like gene within a diverse set of organisms, including many lineages of non-photosynthetic bacteria and archaea (Fig. S4; Supplementary Note 4). The regions surrounding the plastocyanin-like genes within coastal *Pelagibacteraceae* genomes included genes involved in electron transport (e.g. ubiquinol-cytochrome c reductases, multicopper oxidases, cupredoxins), genes to cope with and regulate oxidative stress which frequently occurs during electron transport, and genes to export, chaperon, detoxify, and bind to copper (Fig. S5; Supplementary Note 4). We speculate that the plastocyanin-like genes within coastal *Pelagibacteraceae* genomes are involved in electron transport, although future studies are needed to understand the functional role of these genes within *Pelagibacteraceae* and other non-photosynthetic prokaryotes [65].

#### Cellular stress

Differences in the capacities to respond to osmotic and oxidative stresses were found among offshore and coastal *Pelagibacteraceae* genera. Most offshore genera shared a catalase-peroxidase (*katG*) that may help to cope with oxidative stress and has also been suggested to play a role in the co-evolution of marine microbial communities [15] (Fig. 4, Supplementary file 2F). Coastal genera contained two genes involved in osmotic stress that were not prevalent among offshore genomes: a voltage-gated sodium channel (*vgsc*) and a cation: H + antiporter (*yrbG*) (Fig. 4, Supplementary File 2F). Voltage-gated sodium channels utilize a structurally complex gating system to physically open and close the pore in response to changes in fluid shear or membrane stretch from dehydration [66, 67], and may be particularly useful in coastal Kāneʻohe Bay where spatiotemporal shifts in salinity are common [68].

#### Carbon metabolism

Carbon metabolisms unique to coastal and offshore genera were remarkably distinct. SAR11’s specialization on low-molecular weight, labile carbon sources has likely facilitated the success of this marine oligotroph in open oceans where carbon substrates are limited in availability and under high competition [69–71]. Consistent with these observations, the carbon metabolisms shared by offshore genera did not rely on glycolysis and involved low-molecular-weight compounds (one-carbon to three-carbon; C1–C3) that are common molecules and/or typical waste products in the marine environment. This includes the genetic potential to metabolize organic acids such as D-lactate, oxalate, haloacetate, and formate, as well as diverse aldehydes (Fig. 4; Fig. S6; Supplementary File 2F). In contrast, coastal genera contain gene content to specialize in the degradation of relatively larger carbon compounds (C4–C6), including the sugars D-xylulose and D-ribulose via *xylB* and sugar alcohols arabinitol and mannitol via an arabinitol dehydrogenase, both of which subsequently enter glycolysis through the non-phosphorylative Entner–Doudoroff (ED) pathway. Some coastal groups also have the capacity to metabolize the sugar acids D-tartrate and malate via *tarD* (Fig. 4; Fig. S6; Supplementary File 2F). Genomes belonging to the coastal genus *Undatipelagibacter*, which have the largest genomes among known *Pelagibacteraceae*, harbored gene content potentially involved in the transport and metabolism of glycerophospholipids and sn-glycerol-3-phosphate (*upgEBQ*, *gpr*, *glpA*), the capacity to metabolize L-fucose, D-arabinose, and L-xylose to pyruvate through a L-fucono-1,5-lactonase (*fucB*) and the Entner-Doudoroff (ED) glycolytic pathway, and a fructose transport system (*frcABC*) (Fig. 4; Fig. S6; Supplementary File 2F). The ED glycolytic pathway was core among *Undatipelagibacter*, *Xanthinipelagibacter*, *Alipelagibacter*, and *Accedentipelagibacter* genomes and variable across genomes in other genera (Fig. S6; Supplementary File 2F). The increased prevalence of sugar metabolisms among coastal genera, especially the *Undatipelagibacter* genus, suggests broadened metabolic versatility among coastal *Pelagibacteraceae* in response to a diverse and/or more readily available set of organic carbons found within coastal Kāneʻohe Bay relative to offshore waters. The distinct distributions of carbon metabolisms resolved here also suggests fine-scale niche partitioning along the sugar-acid spectrum within *Pelagibacteraceae* [72].

#### Adhesion

While investigating the metabolic differences between coastal and offshore genera, we observed homologs of a large gene (~5000 to 9500 bp) annotated as an autotransporter adhesin that was almost always present among coastal genomes and absent in all offshore genomes (Fig. 4). Autotransporters provide a simple and relatively minimal mechanism for the delivery of a passenger protein to the surface of Gram-negative bacteria [73], and can vary greatly in size due to changes in the number of repeating sequences in the passenger protein [74]. Because repeating regions cause difficulty for short-read assemblers such as those used in our genome assemblies [22], we further established confidence in the assembly of this gene in our coastal genomes by successfully finding homologs in long-read sequencing libraries from the coastal Kāneʻohe Bay environment and comparing protein structure models of genes annotated from the long-read data to a known autotransporter (Fig. S7; Supplementary Note 5). We examined the genome context of the adhesion gene and found it always positioned next to genes involved in type IV pilus systems and/or type II secretory systems, and sometimes within hyper-variable regions (Fig. S7, Supplementary Note 5, Supplementary File 2F).

Adhesion to surfaces has not been observed in *Pelagibacteraceae* in culture or through environmental studies, although long filaments believed to be pili were observed within dividing cells of *Pelagibacteraceae* [75]. In bacteria, pili are associated with multiple processes, including adhesion to surfaces, transformation competence, and DNA uptake [76, 77]. Although speculative, it is possible that the autotransporter adhesin gene may be involved in the uptake of nutrient or organic carbon in *Pelagibacteraceae* as well [78]. Unfortunately, efforts to identify homologs of the *Pelagibacteraceae* autotransporter adhesin genes in other prokaryotes did not provide further insights into the functional potential of these genes (Fig. S8; Supplementary Note 5). Regardless, the coastal distribution of the autotransporter gene suggests that the advantage of having this large of a gene is worth the trade-off of maintenance in more productive coastal environments, but potentially not in less productive offshore environments.

In summary, our investigation of the distribution of genes related to nutrient and organic carbon acquisition and cellular stress that are unique to coastal and offshore genera reveals numerous subtle differences in metabolic traits driven by the presence or absence of single genes (e.g. high affinity iron transporters, voltage-gated sodium channel genes), but few differences in multi-gene metabolic pathways outside of purine metabolism, molybdenum cofactor biosynthesis, and fucose metabolism. Differences were predominantly related to organic carbon and nitrogen metabolisms, and help to further define the unique and diverse roles *Pelagibacteraceae* play in oceanic biogeochemical cycles. As the distribution of these metabolic traits are better explained by biogeographical patterns than by the phylogenetic relationships of populations that encode them, it is most likely that the metabolic activities they contribute are shaped by environmental pressures.

Our hypotheses regarding the role of the genes we have identified here to cope with nutrient, organic carbon, osmotic, and oxidative stresses assume that they are not specific to populations within KByT, but are a set of “environmental core genes” [26] that are also present in the same *Pelagibacteraceae* populations in coastal and offshore waters elsewhere. Even though habitat-preferences of *Pelagibacteraceae* genera have been established by global metagenomic read recruitment surveys conducted in previous studies [11, 12, 22], such genus-level patterns may not explain gene distribution patterns. To investigate whether the habitat-specific metabolic genes identified within KByT are also present in matching populations in distinct biogeographies, we examined the relationship between the coverage of habitat-specific genes and the coverage of *Pelagibacteraceae* genomes to which they belong across a set of globally distributed coastal and open ocean metagenomes (Supplementary Note 6). We observed highly positive correlations between gene coverages and genome coverages of *Pelagibacteraceae* isolates for most habitat-specific genes (Fig. S9, Supplementary Note 6), confirming that the distribution of the habitat-specific genes generally track the biogeography of the genome. These findings suggest that these genes may be relevant to the niche-partitioning of *Pelagibacteraceae* across coastal and offshore waters at broader geographic scales.

### Gene determinants of habitat-specificity are under relatively higher selective pressures compared to non-diagnostic genes

Our analyses indicated a relatively small number of functions in *Pelagibacteraceae* appear to be associated with its strict niche partitioning. Assuming that the requirement to perform optimally may be higher for genes that determine fitness to particular lifestyles, we hypothesized that metabolic traits that have distinct environmental distributions should be under high selective pressures compared to other genes in the environment. To test this hypothesis, we examined the proportion of non-synonymous to synonymous (pN/pS) sites per gene across *Pelagibacteraceae* genomes using four deeply sequenced metagenomic samples from the KByT system.

To define the set of genomes to be included in this analysis, we first evaluated whether the gene-level selective pressures were uniform across genomes from the same genus. We examined variation in pN/pS across genes with shared KOfam assignments for genomes belonging to the genus *Coralipelagibacter,* which has a low minimum gANI (84.5%, *n* = 6) and is abundant in coastal Kāneʻohe Bay. Gene-to-gene variation explained 82% of the variation and sample-to-sample variation and genome-to-genome variation only explained 0.26 and 0.09%, respectively (Analysis of Variance - ANOVA, Supplementary File 3A). The little genome-to-genome variation showed that genomes within the same genus likely experience similar patterns of selection on shared genes, and supported using a single genome as a representative per genus. Thus, our downstream analyses utilized a single genome for each genus in *Pelagibacteraceae*, except *Atlantikopelagibacter*, *Pelagibacter*, *Accedentipelagibacter*, and *Alipelagibacter*, which lacked sufficient coverage in the KByT system (Fig. S3, Supplementary File 3B).

Consistent with previous observations [18], strong purifying selection (pN/pS ≪ 1) was a general characteristic of most genes across the examined *Pelagibacteraceae* genomes [18], where over 75% of genes in each genome representative had a pN/pS value ≤0.1 (Supplementary File 3C). Gene-to-gene variation in pN/pS values explained between 88% and 99% of the variation in pN/pS (ANOVA, Supplementary File 3D), showing that some genes were under higher selective pressures than others. Metagenomic sample and gene coverage both explained a very small proportion of the pN/pS variation within genomes (<0.5% and < 0.07%, respectively; ANOVA; Supplementary File 3D). Gene coverage showed no correlation between per gene values of pN/pS (absolute value of *r* = 0.02–0.28; Pearson correlation coefficient; Fig. S10), which indicates that pN/pS values are unlikely to be driven by artifacts associated with variation in coverage.

**Figure 5:**
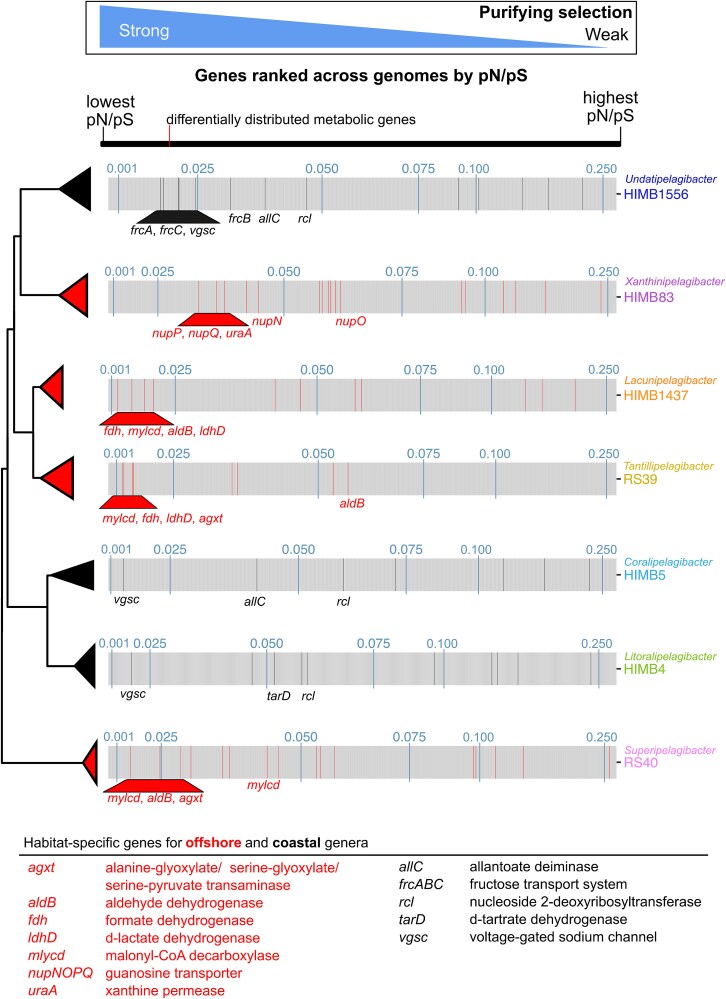
Habitat-specific genes are under relatively higher selective pressures compared to non-diagnostic genes. Across each genome, pN/pS values per gene are ranked from lowest pN/pS value (high purifying selection) to highest pN/pS value (low purifying selection). Genes that were characterized as differentially distributed between offshore and coastal *Pelagibacteraceae* genera in Fig. 4 are colored red (offshore) or black (coastal) across the genomes. The gene names are noted and the description of the gene is provided in the key for genes that are discussed in the text. The full suite of gene names and descriptions can be found in Fig. S11. pN/pS values of 0.01, 0.025, 0.05, 0.075, 0.1, and 0.25 are shown in blue and genomes are ordered by their phylogenomic relationships. pN/pS: Proportion of non-synonymous to synonymous.

We next evaluated the selective pressures on genes that were differentially distributed across coastal and offshore *Pelagibacteraceae* genera (Fig. 5; Fig. S11). The differentially distributed genes with the highest selective pressures in offshore genera included a malonyl-CoA decarboxylase (*mlycd,* pN/pS = 0.019 ± 0.010, mean ± sd), an aldehyde dehydrogenase (*aldB*, pN/pS = 0.033 ± 0.018), a formate dehydrogenase (*fdh,* pN/pS = 0.0127 ± 0.001), a D-lactate dehydrogenase (*ldhD*, pN/pS = 0.018 ± 0.003), and an alanine-glyoxylate / serine-glyoxylate / serine-pyruvate transaminase (*agxt*, pN/pS = 0.022 ± 0.008) (Fig. 5). These genes are generally involved in the metabolism of small and common carbon substrates and the capacity to produce glycine and serine, which are auxotrophies in other *Pelagibacteraceae*. Within *Xanthinipelagibacter*, genes involved in purine uptake (*uraA*, pN/pS = 0.040 ± 0.001; *nupNOPQ*, pN/pS = 0.045 ± 0.010) experienced higher purifying selection than purine degradation pathways (*xdhAB*, pN/pS = 0.084 ± 0.028), which is in line with previous observations that high-affinity transport is key to *Pelagibacteraceae*’s success as an oligotroph [79, 80]. The low pN/pS values for these distinct carbon and nitrogen related genes support the hypothesis that these metabolisms provide a fitness advantage in offshore waters.

Among genes unique to coastal genera, the voltage-gated sodium channel was under high selective pressure (*vgsc*, pN/pS = 0.016 ± 0.004). Coastal Kāneʻohe Bay harbors a larger range of salinity, temperature, and pH conditions compared to the adjacent offshore [40, 54, 68], and thus the voltage-gated sodium channels may be critical for coastal genera as they rapidly regulate ionic composition [66]. The allantoin deiminase (*allC*, pN/pS = 0.039 ± 0.002) and nucleoside 2-deoxyribosyltransferase (*rcl*, pN/pS = 0.054 ± 0.006) were also under moderately high selective pressures in coastal genomes, further supporting the specialization of coastal genera on allantonate and nucleotides and a separation in purine metabolism niche from offshore genera. Three sugar transporter genes (*frcABC*, pN/pS = 0.024 ± 0.006) within *Undatipelagibacter* and a tartrate dehydratase (*tarD*, pN/pS = 0.051 ± 0.001) within *Litoralipelagibacter* exhibited low pN/pS values suggesting that sugar metabolisms and transporters are not only more common genomic features in the coastal genera, but also likely essential for their fitness.

In order to identify the *Pelagibacteraceae* core genes under the highest selective pressures in the KByT system, we examined the mean pN/pS values for core genes found across the same seven genomes. The majority of core genes were under high selection (pN/pS < 0.1; *n* = 474 of 612 core genes). Genes involved in energy production and conversion, translational, ribosomal structure and biogenesis, transcription, amino acid transport and metabolism, and posttranslational modification, protein turnover, and chaperones were common among genes under high selective pressures (Fig. S12, Supplementary File 3E, Supplementary Note 7). Two genes involved in transcriptional regulation of responses to acidity and glycine were among the top core genes with the most purifying selection (pN/pS 0.019 ± 0.011; 0.02 ± 0.01, respectively). The low pN/pS values for these genes supports that although *Pelagibacteraceae* may have few regulatory genes generally [56], the ones it does have likely impact the performance and survival of *Pelagibacteraceae* cells.

Among the core genes under the highest purifying selection, were both subunits of an adenylylsulfate reductase (*aprAB*: mean pN/pS 0.018 ± 0.005 and 0.018 ± 0.008, respectively). Adenylylsulfate reductases are involved in dissimilatory sulfur metabolism through the interconversion of adenylyl sulfate (APS) to sulfite. Consistent with previous analyses [81], incomplete dissimilatory and assimilatory sulfate reduction pathways were core among all *Pelagibacteraceae* examined in this study (Fig. S13). As previously reported [82], adenylylsulfate reductase likely removes sulfite that accumulates during the degradation of organic sulfur compounds. Further supporting this hypothesis, we found multiple core or near core genes involved in the production of sulfite from reduced organosulfur compounds: (2R)-sulfolactate sulfo-lyase (*suyAB*), a sulfoacetaldehyde acetyltransferase (*xsc*), and a sulfur dioxygenase (Fig. S13). Our analyses reveal the genetic potential to metabolize a wide diversity of reduced organosulfur compounds as alternative sources of sulfur and that adenylylsulfate reductases (*aprAB*) likely plays an important role in accommodating *Pelagibacteraceae*’s unique sulfur metabolisms.

## Discussion

Through genomic and metagenomic characterizations of a recently expanded collection of SAR11 isolates [22], our study reveals a polyphyletic distribution of coastal ocean versus offshore habitat specialists among closely related SAR11 populations, where the emergence of key genetic features that underlie these strong habitat-preferences appear to have occurred through independent evolutionary events rather than transfer from a single common ancestor. Genera that share the same environment (e.g. coastal or offshore) have also accumulated shared and unique gene content that is predominantly involved in the metabolism of organic carbon and the acquisition of nitrogen from organic sources. A subset of these metabolic genes are under high purifying selection, emphasizing the importance of these functions to the fitness of distinct *Pelagibacteraceae* genera and highlighting potential determinants of niche differentiation of *Pelagibacteraceae* in coastal and offshore environments.

The cohesion of both ecological and genetic diversity observed within genera of *Pelagibacteraceae* most closely resembles ecotypes: ecologically homogeneous groups of closely related bacteria whose genetic diversity is guided by cohesive forces of selection, recombination, and genetic drift [83, 84]. Ecotypic differentiation has previously been suggested to describe the spatial and temporal variation partitioning genetic and genomic SAR11 diversity [9, 12, 22, 54, 85–90], however the underlying evolutionary processes or functional differences contributing to this diversification have rarely been linked to patterns of distribution. Our findings reveal evolutionary drivers behind ecotypic differentiation by explaining distinct ecological distribution patterns with differences in gene content, metabolic potential, and selective pressures across ecotypes. The use of a constrained model system that provided both metagenomic sequence data as well as isolate genomes from local *Pelagibacteraceae* populations was paramount to being able to resolve signatures of environmental selection in sympatric and parapatric SAR11 populations. The power of this approach is further demonstrated in the fact that most of the *Pelagibacteraceae* ecotypes defined in this constrained system as having preferences for coastal oceans or the open ocean appear to hold when global read recruitment data were examined [22].

Our results suggest that *Pelagibacteraceae* has transitioned between offshore and coastal environments multiple times, with a handful of genes that support these lifestyles likely acquired through multiple independent processes. The small number of adaptive genes are shared among ecologically similar, but polyphyletically distributed genera, where genomes have maintained a substantial amount of genetic diversity. Given the distribution patterns of the adaptive genes and the genetic diversity maintained within the populations that carry them, the proliferation of adaptive genes unlikely occurred via genome-wide selective sweeps [53], a process that describes the clonal expansion of an individual subpopulation that carries an adaptive gene leading to a genome-wide reduction in genetic diversity. Instead, the maintenance of genetic diversity and the relatively strong purifying selection to maintain the functional identity of these adaptive genes is consistent with gene-specific selective sweeps [91–95].

Consistent with previous studies that have identified a handful of genes responsible for habitat-specific adaptation [26, 93, 95], the gene content differences attributed to stable ecological speciation here are generally found within the relatively stable genomic backbone, rather than within hyper-variable genomic islands. This is a critical observation as it links eco-evolutionary processes to specific portions of the immense genomic diversity that exists within *Pelagibacteraceae*, and underscores the importance of analyses informed by genomic architecture. Horizontal gene transfer (HGT) events, a likely pathway for the acquisition of the genes driving habitat-specific adaptation in *Pelagibacteraceae*, can target different portions of the genome, including but not limited to hyper-variable genomic islands. Although the original HGT event may have occurred within an existing variable region or created a new region of variability, subsequent gene sweep events driven by the newly acquired genes could stabilize the surrounding genomic context, ultimately incorporating genes in variable regions into the genomic backbone of an environmental population. Future studies that examine the contribution of gene content found within hyper-variable islands to stable ecological speciation as opposed to incipient speciation or intraspecific diversity would provide a better understanding of the role of hyper-variable regions in long-term evolutionary processes.

Our study illuminates evolutionary processes that underlie the niche differentiation of sympatric and parapatric populations of marine bacteria, and points to gene sweeps driven by environmental selection and metabolic specialization as the drivers of habitat-specificity in SAR11. This work broadens the metabolic diversity known from *Pelagibacteraceae* genomes, suggesting that metabolic versatility has contributed to SAR11’s success in the global ocean. Although metabolic reconstructions are subject to uncertainties that necessitate physiological exploration with controlled experimentation in the laboratory, the metabolic analysis of high-quality genomes from cultivated isolates provides the opportunity to delineate metabolic specialization that could advance cultivation approaches and allow for more targeted media recipes [96–99]. SAR11 is estimated to oxidize between 6% to 37% of gross ocean primary productivity [20]. Future work combining these metabolic predictions with *in situ* measures will greatly improve our understanding of how SAR11 impacts the dissolved organic matter pool and biogeochemical cycles in the ocean.

## Supplementary Material

Revised_Supplementary_file_1_Tucker_wraf216

Revised_Supplementary_file_2_Tucker_wraf216

Revised_Supplementary_file_3_Tucker_wraf216

tucker_ISMEJ_SM_Sep192025_wraf216

## Data Availability

All data are available in the main text or the Supplementary Materials. Accession information for genome sequences used to conduct pangenomic analyses and read recruitment in this study are found in Supplementary File 1a. Short-read metagenome accession IDs and environmental data used in this study are provided in Supplementary File 1c. Long-read metagenomes from coastal Kāneʻohe Bay are available under NCBI Project PRJNA1201851. Code to run analyses and create figures can be found at https://github.com/tucker4/SAR11_metabolism.
