# Supplementary material for: Habitat-specificity in SAR11 is associated with a few genes under high selection
Source: ISME J. 2025 Oct 11;19(1):wraf216. doi: 10.1093/ismejo/wraf216 (PMC12636525; doi:10.1093/ismejo/wraf216)
Supplement: tucker_ISMEJ_SM_Sep192025_wraf216 [file tucker_ismej_sm_sep192025_wraf216.pdf]

Supplementary Materials for:

Habitat-specificity in SAR11 is associated with a few genes under high selection

Sarah J. Tucker *et al.*

Emails of corresponding authors: meren@hifmb.de, rappe@hawaii.edu

This PDF file includes:

Supplementary Text
Figs. S1 to S13
Legends for Supplementary files 1 to 3

Other Supplementary Materials for this manuscript include the following:

Supplementary files 1 to 3

Supplementary Text

Supplementary Note 1: K-means clustering of biogeochemical data

K-means analysis of surface ocean biogeochemical parameters from 192 surface ocean samples from KByT and Station ALOHA revealed two clusters. These were termed a coastal cluster (n=92) and an offshore cluster (n=100) based on the biogeochemical characteristics and source location of the samples. Environmental covariates underlying the cluster delineation were mapped using a Principal Components Analysis (PCA) that explained 45.1% and 14.7% of the variation across PC1 and PC2, respectively (**Fig. S2**).

Supplementary Note 2: Descriptions of *Pelagibacteraceae* metabolic traits

Core metabolic traits of *Pelagibacteraceae*

Consistent with previous studies, an incomplete Embden-Meyerhof-Parnas glycolytic pathway, an incomplete oxidative portion of the pentose phosphate shunt, a complete non-oxidative portion of the pentose phosphate shunt, a complete tricarboxylic acid (TCA) cycle, incomplete assimilatory and dissimilatory sulfate reduction pathways, and a gene encoding bacteriorhodopsin were core to the *Pelagibacteraceae* genera examined here (**Supplementary file 2h**) [1–3]. Based on the metabolic machinery available, other sources of carbon core to *Pelagibacteraceae* likely include the use of malate, L-lactate, glycolate, pyruvate, methanol, acetaldehyde, and formate (**Supplementary file 2h**).

All *Pelagibacteraceae* genomes had the gene repertoire to synthesize 18 of the 20 essential amino acids and no genome could synthesize the 21st protogenetic amino acid, selenocysteine (**Supplementary file 2h**). Most *Pelagibacteraceae* genomes are missing genes in the pathway for the biosynthesis of amino acids glycine and serine (**Supplementary file 2h**), and

although all genomes contain a threonine aldolase which could potentially complete the glycine biosynthesis pathway, growth experiments with *Pelagibacteraceae* isolates show that threonine aldolase did not satisfy cellular glycine demands [4]. As an exception to the serine and glycine auxotrophies found in most *Pelagibacteraceae*, genomes belonging to *Tantillipelagibacter*, *Superipelagibacter*, and *Altantikopelagibacter* contain an alanine-glyoxylate transaminase / serine-glyoxylate transaminase / serine-pyruvate transaminase (**Supplementary file 2h**), which provides an alternative and complete pathway for glycine and serine biosynthesis. Full biosynthetic pathways for 4-aminobenzoic acid (PABA) and riboflavin (vitamin B2) biosynthesis were core to all the *Pelagibacteraceae* genera but the pathways for thiamine (vitamin B1), pantothenate (vitamin B5), pyridoxine (vitamin B6), biotin (vitamin H), cobalamin (vitamin B12), niacin (vitamin B3), folate (vitamin B9), nicotinamide adenine dinucleotide (NAD), and myoinositol biosynthesis were not complete in any of the genera (**Supplementary file 2h**). Since methionine is a costly amino acid to synthesize de novo, methionine salvage would provide an advantage for *Pelagibacteraceae*. We observe an incomplete Yang cycle for methionine salvage, however, we predict that the dihydroxyacetone phosphate (DHAP)-methanethiol shunt [5] may provide a salvage pathway for methionine (**Supplementary file 2h**).

Pelagibacteraceae is well known for having incomplete assimilatory and dissimilatory sulfate reduction pathways [6]. However, the variety of organosulfur metabolisms available to utilize reduced sulfur sources and the distribution of the genes across *Pelagibacteraceae* genera are not well understood. Our analyses revealed that all *Pelagibacteraceae* genomes have the capacity to metabolize 2,3- dihydroxypropane-1-sulfonate (DHPS), sulfoacetaldehyde, sulfolactate, and thiosulfate for reduced forms of sulfur and/or carbon (**Fig. S7, Supplementary file 2h**). Dimethylsulfoniopropionate (DMSP) can be catabolized via the demethylation pathway

by all genera, and a subset of genera and genomes can also metabolize taurine, alkanesulfonates, and methanethiol. All *Pelagibacteraceae* can remove sulfite created from many of these organosulfate metabolisms from the cell by turning it into adenylyl sulfate (APS) via an adenylylsulfate reductase (*aprAB*). Among the sulfur-related acquisition genes, cysteine-O-acetylserine efflux proteins (*eamA* and/or *eamB*), s-adenosylmethionine transporters, taurine (and sulfonate) transporters *tauABC*, and sulfite exporter (*tauE*) were core (**Supplementary file 2h**). In addition, we found that all *Pelagibacteraceae* genomes can oxidize sulfur in the presence of glutathione through a sulfur dioxygenase.

Metabolic traits that varied across genera

Some central carbon metabolisms were highly varied among and within *Pelagibacteraceae* genera, with few clear patterns associated to ecology (e.g. coastal versus offshore) or evolutionary history, including the Enter-Doudoroff (ED) glycolytic pathway, a phosphoenolpyruvate carboxylase, and the glyoxylate bypass (**Supplementary file 2h**). The ED glycolytic pathway was found in some but not all *Pelagibacter* (5 of 7, HTCC1002, HTCC1013, HTCC1040, HTCC1062, NP1) and *Coralipelagibacter* genomes (2 of 6, HIMB5 and HIMB1564) and all *Undatipelagibacter*, *Xanthinipelagibacter*, *Alipelagibacter*, and *Accedentipelagibacter* genomes (**Supplementary file 2h**). The presence of the ED glycolytic pathway opens up a variety of sugar and sugar acid metabolisms to select *Pelagibacteraceae*, with extra investment in related gene content from *Undatipelagibacter*, *Xanthinipelagibacter*, and some *Accedentipelagibacter* (**Supplementary file 2h**). Some genomes from *Undatipelagibacter* and *Xanthinipelagibacter* have gene content to metabolize fucose and/or arabinose, a specialized transporter for xylose, and epimerases for fucopyranoses and aldoses.

Xanthinipelagibacter and *Undatipelagibacter* also uniquely shared a beta-glucosidase (EC 3.2.1.21), an alpha-galactosidase (EC 3.2.1.22), and a raffinose/stachyose/melibiose transport gene, suggesting at least partial metabolism of these compounds (**Supplementary file 2h**).

All genomes from genera *Undatipelagibacter*, *Coralipelagibacter*, *Accedentipelagibacter*, *Xanthinipelagibacter*, *Tantillipelagibacter*, *Atlantikopelagibacter*, *Superipelagibacter* and half of the genomes from *Pelagibacter* have a phosphoenolpyruvate carboxylase (EC 4.1.1.31), which produces an anaplerotic reaction that assimilates CO_2 from glycolysis products to oxaloacetate entering the TCA cycle (**Supplementary file 2h**). The glyoxylate bypass is akin to a modified TCA cycle but prevents the loss of carbon and thus supports growth on acetyl-CoA or products that were turned to acetyl-CoA as a sole carbon source. The glyoxylate bypass was conserved across most genera, however more than half of the genomes from the genus *Xanthinipelagibacter* (HIMB1409, HIMB1413, HIMB1444, HIMB2201, HIMB2250) and two genomes from *Undatipelagibacter* (HIMB1573, HIMB1685) were missing an isocitrate lyase, *aceA* (**Supplementary file 2h**). Genera *Xanthinipelagibacter*, *Undatipelagibacter*, *Atlantikopelagibacter*, *Accedentipelagibacter*, and *Coralipelagibacter* and HIMB1527 in the *Litoralipelagibacter* genus have the ability to metabolize the common phytoplankton osmolyte trigonelline to formate, succinate semialdehyde, and methylamine (**Supplementary file 2h**). Carbon monoxide metabolism varied among genera with *Coralipelagibacter*, *Litoralipelagibacter*, *Alipelagibacter*, and some *Xanthinipelagibacter* (3 out of 8) (**Supplementary file 2h**). Some *Undatipelagibacter*, *Xanthinipelagibacter*, and *Accedentipelagibacter* genomes can also metabolize ribose and D-arabinonate.

HIMB83 in the genus *Xanthinipelagibacter* and HTCC7211 and HTCC7217 from *Atlantikopelagibacter* had phosphonate transporters (*phnCDE*) (**Supplementary file 2h**).

HTCC7211 and HTCC7217 from *Atlantikopelagibacter* (2 of 3 genomes) had the ATPase component of the phosphate transporter, *phnK*. HTCC7211 and HTCC7217 also had *phnGHIJLMP* to degrade methylphosphonates to products entering the pentose phosphate pathway and *phnX* (EC 3.11.1.1) to degrade phosphonacetaldehyde to acetaldehyde and orthophosphate. The prevalence of phosphonate pathways in *Atlantikopelagibacter*, as well as various other metabolic genes unique to this genus, like the methanethiol oxidase (EC:1.8.3.4), a nitroreductase, and formaldehyde metabolism (**Supplementary file 2h**), highlight potential differences between metabolisms of open-ocean *Pelagibacteraceae* in the Atlantic, where *Atlantikopelagibacter* genomes were isolated, and those in the Pacific.

Supplementary Note 3: Analysis of molybdenum cofactor enzyme loss

There are two types of molybdenum cofactors: iron-molybdenum cofactor (FeMoco) which gives rise to molybdenum nitrogenases and molybdenum enzymes with a pterin cofactor [7]. Based on the distribution of molybdenum cofactor biosynthesis genes and genes involved in metabolisms that require a molybdenum cofactor (e.g. carbon monoxide dehydrogenases, xanthine dehydrogenases, etc), we hypothesized that molybdenum cofactors were lost from the *Tantillipelagibacter* and *Lacunipelagibacter* lineages. We also evaluated the distribution of iron-molybdenum cofactor genes. While some *Pelagibacteraceae* contain a number of the biosynthetic genes which are needed to form the iron-sulfur cluster of nitrogenases (e.g. *nifU*, *nifB*, and/or *nifS*), none have *nifE* or *nifN*, which are needed to stabilize the cofactor to receive the molybdenum, nor *nifK, nifD,* or *nifH*, which form the catalytic portion of the nitrogenase (**Supplementary file 2d**) [7]. Thus we find it unlikely that *Pelagibacteraceae* in this genome collection contain iron-molybdenum cofactor nitrogenases.

Supplementary Note 4: Plastocyanin-like homolog search and genomic context

We examined homologs of the plastocyanin-like genes found in coastal *Pelagibacteraceae* genomes to help contextualize the potential functional role of this gene in *Pelagibacteraceae*. We used the anvi-search-functions program in anvi'o v8.0 [8] to export the amino acid sequences of the plastocyanin-like genes from the *Pelagibacteraceae* genomes. Next, we used protein sequences clustered at 40% identity from the GlobDB release 226 [9] database. A total of 8,746 protein sequence representatives had annotations to KEGG Ortholog ID K02638 (plastocyanin; *petE*). We used the makeblastdb command from BLAST [10] -dbtype prot flag to create a blast database of the GlobDB sequences. With the search sequence set, we blasted the sequences from the *Pelagibacteraceae* genomes against the database of using the blastn function with an e-value of 1e-10 and the flag -max_target_seqs 9000, and subsequently filtered the blast hit results to only include matches that had a minimum of 70% coverage of the genes from our genome's sequences. We then aligned the sequences with outgroups, including two plant (InterPro Gene IDs A0A178WAZ3 and A0A022PRV7) and two eukaryotic phytoplankton (InterPro Gene ID XP_002504072; NCBI Gene ID XRB07048.1) plastocyanin sequences, using muscle [11] and estimated a tree using using IQ-Tree v2.12 [10] with 1000 ultrafast bootstraps and the LG+F+R10 model. The tree was rooted with the outgroup sequences within an anvi-interactive session of anvi'o v8.0 [8].

Cyanobacteria and the eukaryotic phytoplankton represent groups where the functional role of plastocyanins are well established as an electron carrier from cytochrome *f* to photosystem I [12, 13]. In the phylogeny (**Fig. S4**), sequences assigned to cyanobacteria were positioned closer to the eukaryotic sequences than the remaining sequences from non-photosynthetic prokaryotes. The three sequences from coastal *Pelagibacteraceae* grouped with

other *Pelagibacterales* sequences from GlobDB, including sequences from isolate HTCC9565 (genus *Pelagibacter*) and six *Pelagibacterales* environmental genomes. However, sequences from diverse taxonomic orders (e.g. *Magnimaribacterales*, *Rhodospirillales*) also grouped with the *Pelagibacterales* sequences. Overall, the lack of taxonomic clustering below the phylum level is indicative of a weak phylogenetic signal within the *petE* gene and its homologs, suggesting it is not possible to assess close relatives of the petE sequences from coastal *Pelagibacteraceae*. Weak phylogenetic signal within bacterial petE sequences was observed previously [12]. These results show that plastocyanin-like sequences can be detected within multiple clades of non-photosynthetic bacteria, and future research is needed to address the evolution, ecology, and function of these genes outside of photosynthetic lineages.

We next looked at the genomic context surrounding the plastocyanin-like genes in coastal *Pelagibacteraceae* genomes using edited output from anvi-summarize from anvi'o v8.0 [8] as input into gggene v 0.5.0 (https://wilkox.org/gggenes/). The regions surrounding the plastocyanin-like genes contained a high number of genes involved in electron transport (e.g. ubiquinol-cytochrome c reductases, multicopper oxidases, cupredoxins), genes to cope with and regulate oxidative stress (which frequently occurs during electron transport), and genes to export, chaperone, detoxify, and bind to copper (**Fig. S5**). Thus, we speculate that the plastocyanin-like genes within *Pelagibacteraceae* are involved in electron transport and contain copper.

Supplementary Note 5: Homolog searches with fibronectin autotransporter adhesin gene

To ensure the length and quality of autotransporter adhesion gene calls in *Pelagibacteraceae* genomes, we first examined the genome context and length of the gene calls matching to the fibronectin autotransporter adhesin (K19231) and/or its associated COG20

functions (COG3468, COG3210) in the genomes. Two genomes (HIMB1623 and HIMB1709) were excluded from this analysis because they contained genes annotated as fibronectin autotransporter adhesins that were located at the very beginning or end of contigs and were much larger in size than those from other genomes (>10,000 bp). Another five genomes from the genus *Pelagibacter* contained homologs to fibronectin autotransporter adhesin genes, but because the genus *Pelagibacter* is not frequently detected in the tropical Pacific, these genomes were also removed from this analysis. DNA sequences of the remaining autotransporter adhesion genes found within the *Pelagibacteraceae* genomes were used to make a blast database with the makeblastdb command from BLAST [10].

Second, we searched for gene homologs in metagenomes collected from surface seawater samples taken from coastal Kāneʻohe Bay and sequenced using long-read (PacBio) sequencing technologies [14]. After converting four long-read sequence libraries from a fastq file format to a fasta file using seqtk (https://github.com/lh3/seqtk), contig databases were built in anvi'o v8.0 [8] and genes called and annotated with Clusters of Orthologous Groups (COG) as described in the main methods section. We used the anvi-search-functions program to export the DNA and amino acid sequences of the genes of interest (COG3210, COG3468) annotated in the long-read samples. Next, we queried the autotransporter adhesin gene sequences exported from the long-read metagenomes against the database of autotransporter adhesion genes found in the genomes with a blastn function using an e-value of 1e-10. The resulting matched sequences from the long-read metagenomes were quality filtered based on high sequence identity (>90%) and subsequently for near exact matches, where the alignment of the DNA sequence from genes from the long-read sequences covered nearly the whole gene in the genome (±100 bp).

The amino acid sequences for near exact matches in the long-read sequencing libraries, the amino acid sequences from the genomes, and three outgroup sequences (NCBI-ProteinIDs: ACH51883, AZY97442, JW1503) were aligned using the slow/accurate setting of Clustal W 2.1 (https://www.genome.jp/tools-bin/clustalw). A tree was estimated using IQ-Tree v2.12 [15] with 1000 ultrafast bootstraps and the best fit model was chosen with ModelFinder [16]. Using amino acid sequences for select near exact matches, we estimated 3-D protein structure models using ColabFold v 1.5.2 [17] on the COSMIC^2 science gateway [18]. The results were visualized on RCSB (https://www.rcsb.org/3d-view) [19] using the Mol* viewer [20]. The 3D visualizations were compared against known structures of autotransporters [21].

Homolog searches to examine autotransporter adhesin genes in long-read sequencing data recovered nine non-redundant gene sequences that were near exact matches to most gene sequences in *Pelagibacteraceae* genomes (33 of 43; **Fig. S7**). Protein structure models of this gene shared characteristics expected of autotransporter proteins: β-barrel translocator domain, α-helical linker, and passenger domains containing β helices [21] (**Fig. S7**). In coastal *Pelagibacteraceae* the autotransporter was located in hypervariable regions within some genomes, but not others, and was always positioned next to genes involved in type IV pilus systems and/or type II secretory systems (**Fig. S7**).

To identify homologous genes in other microorganisms, we exported all sequences matching to KEGG ortholog K19231 (fibronectin-binding autotransporter adhesin; n=1135) from the set of clustered proteins from the GlobDB release 226 [9]. We used this set of search sequences to make a blast database with the makeblastdb command from BLAST [10] with the -dbtype prot flag. We then blasted the exported amino acid sequences of fibronectin-binding autotransporter adhesin from the *Pelagibacteraceae* genomes against the database using the

blastn function. The BLAST hit results were subsequently filtered to only include matches that had a minimum of 70% coverage of the genes from our genomes and to remove sequences with unexpectedly long sequences (>10,000 AA). The remaining 272 sequences and two outgroup sequences (NCBI GenBank: AKB89180; AYG18348) were aligned using muscle [11] with the flags -maxiters 1 -diags1 -sv to accommodate the long length of the sequences and a tree was estimated using IQ-Tree v2.12 [10] with 1000 ultrafast bootstraps and the LG+F+R10 model. The tree was visualized in anvi'o v8 [8] and rooted at the outgroups.

Examining the fibronectin-binding autotransporter adhesin gene tree, coastal *Pelagibacteraceae* sequences group together on a long branch, while a single sequence from *Fontibacterium* (order *Pelagibacterales*) grouped separately (**Fig. S8**). The nearest neighbors to the coastal *Pelagibacteraceae* fibronectin-binding autotransporter adhesin genes derive from multiple different phyla (i.e. *Planctomycetota*, *Verrucomicrobiia*). A lack of taxonomic clustering at high taxonomic levels suggests a weak phylogenetic signal and prevents drawing conclusions about the close homologs of *Pelagibacteraceae* fibronectin-binding autotransporter adhesin genes.

Supplementary Note 6: Presence of habitat-specific genes in global metagenomes

To ascertain whether genes that were identified as potential determinants of habitat-specificity in *Pelagibacteraceae* in coastal Kāne'ohe Bay and the adjacent offshore are present in coastal and offshore environments of the global ocean, we recruited reads from a small subset of metagenomes (n=16; **Supplementary file 2i**) to select genomes of each *Pelagibacteraceae* genus present in the KByT system (n=8). First, we utilized the coverage results from Freel et al., [22] to identify metagenomes in which the target *Pelagibacteraceae* genomes were abundant and that represent broadly distributed geographic locations (**Supplementary file 2i**). Next, we conducted read recruitment to the isolate genomes following pipelines previously described in the main materials and methods. Briefly, metagenomes were competitively mapped with Bowtie2 v 2.3.5 [23] to an anvi'o contig database of the eight *Pelagibacteraceae* genomes using anvi'o v 8.0 [8]. The anvi-profile function stored coverage and detection statistics of each *Pelagibacteraceae* genome found in the metagenomic samples and the anvi-summarize function with the --init-gene-coverages flag exported these results. The read recruitment for each genome was divided across the total *Pelagibacteraceae* read recruitment for a given sample to yield a relative estimate of each genome within a sample. In R v 4.4.1 [24], we next subsetted the read recruitment results to focus on non-outlier gene coverages of the set of genes identified in **Fig. 4** as potentially contributing to habitat specificity in the *Pelagibacteraceae*. Pearsons correlations between genome coverage (mean Q2Q3 coverage) and non-outlier gene coverages of the habitat specific genes were conducted using the cor.test( ) in the stats base package of R with a log+1 transformation for both gene and genome coverages. The results were then visualized using ggplot2 v 3.5.1 [25].

The coastal and offshore habitat preferences of *Pelagibacteraceae* genera observed within the KByT system have also been detected in other ocean regions [22, 26, 27]. Our read recruitment to a subset of widely distributed samples reflect these findings: HIMB4 (*Litoralipelagibacter*) had high relative abundance within coastal waters of the Mediterranean Sea and Portugal and the Chesapeake Bay, and was largely absent from the oceanic samples (**Supplementary file 2i**), HIMB5 (*Coralipelagibacter*) had low relative abundance across most samples outside of KByT, but increased in relative abundance in waters of coastal Panama and the Red Sea (**Supplementary file 2i**), and HIMB1556 (*Undatipelagibacter*) was abundant in both coastal (e.g. coastal Panama and the Mediterranean Sea) and offshore (e.g. Arabian Sea) waters. Genera identified as offshore specialists within KByT increased in relative abundance in oceanic samples (**Supplementary file 2i**).

We next sought to understand whether the habitat specific genes we identified as playing a critical role in determining coastal or offshore habitat preferences within KByT could be observed in *Pelagibacteraceae* populations in other oceanic regions. Across broadly distributed coastal and open oceans, we observed positive and statistically significant correlations between gene coverage of the habitat specific genes and genome coverage (**Fig. S9**). This finding establishes that the habitat specific genes identified from KByT are commonly found within *Pelagibacteraceae* populations from various ocean provinces, and suggests that these genes may also contribute to coastal and offshore distributions of *Pelagibacteraceae* populations beyond KByT.

Supplementary Note 7: Common functional categories in *Pelagibacteraceae* core genes under high selective pressures

To examine core genes under high selective pressures and the functional categories of these genes, we first examined the distribution of pN/pS values across the core genes (**Fig. S12**). Most genes had low pN/pS values (0.072 ±0.04 pN/pS; mean±sd; n=612). To evaluate the functional categories common in genes with pN/pS, we utilized the COG categories associated with the KOfam gene calls and analyzed the composition of these COG categories across variable pN/pS thresholds. The aim of examining multiple thresholds is to not set a single arbitrary threshold but to show patterns that are relevant across different thresholds. We examined the rank of COG categories across the pN/pS threshold of <0.025, <0.05, <0.1, and across all genes (**Fig. S12**). A small subset of core genes had annotations to more than one COG category: 546 matched to a single COG category, 62 genes matched to two COG categories, and 4 genes matched to three COG categories. Genes annotated to more than one COG category were counted multiple times (**Supplementary file 3e**).

We ranked each COG category within each of the four pN/pS thresholds, where a COG category was ranked as number one if the COG category had the highest count of genes contributing to any COG category at that pN/pS threshold. Subsequently, we visualized results using the package ggbump (https://github.com/davidsjoberg/ggbump) in R v 4.4.1 [24]. We observed consistency within the top ranked COG categories at the two lowest thresholds of pN/pS values, such that energy production and conversion (C), translation, ribosomal structure and biogenesis (J), transcription (K), posttranslational modification, protein turnover, and chaperones (O), and amino acid transport and metabolism (E) were among the top ranked COG categories at both pN/pS <0.025 and pN/pS <0.05 (**Fig. S12**).

References

1. Grote J, Thrash JC, Huggett MJ, et al. Streamlining and core genome conservation among highly divergent members of the SAR11 clade. *MBio* 2012;**3**. 10.1128/mbio.00252-12
2. Eiler A, Mondav R, Sinclair L, et al. Tuning fresh: radiation through rewiring of central metabolism in streamlined bacteria. *ISME J* 2016;**10**:1902–1914. 10.1038/ismej.2015.260
3. Giovannoni SJ. SAR11 bacteria: The most abundant plankton in the oceans. *Ann Rev Mar Sci* 2017;**9**:231–255. 10.1146/annurev-marine-010814-015934
4. Tripp HJ, Schwalbach MS, Meyer MM, et al. Unique glycine-activated riboswitch linked to glycine-serine auxotrophy in SAR11. *Environ Microbiol* 2009;**11**:230–238. 10.1111/j.1462-2920.2008.01758.x
5. North JA, Wildenthal JA, Erb TJ, et al. A bifunctional salvage pathway for two distinct S-adenosylmethionine by-products that is widespread in bacteria, including pathogenic *Escherichia coli*. *Mol Microbiol* 2020;**113**:923–937. 10.1111/mmi.14459
6. Tripp HJ, Kitner JB, Schwalbach MS, et al. SAR11 marine bacteria require exogenous reduced sulphur for growth. *Nature* 2008;**452**:741–744. 10.1038/nature06776
7. Schwarz G, Mendel RR, Ribbe MW. Molybdenum cofactors, enzymes and pathways. *Nature* 2009;**460**:839–847. 10.1038/nature08302
8. Eren AM, Kiefl E, Shaiber A, et al. Community-led, integrated, reproducible multi-omics with anvi'o. *Nat Microbiol* 2021;**6**:3–6. 10.1038/s41564-020-00834-3
9. Speth DR, Pullen N, Aroney STN, et al. GlobDB: A comprehensive species-dereplicated microbial genome resource. *arXiv[q-bioGN]* 2025: arXiv:2506.11896. 10.48550/arXiv.2506.11896
10. Camacho C, Coulouris G, Avagyan V, et al. BLAST+: architecture and applications. *BMC Bioinformatics* 2009;**10**:421. 10.1186/1471-2105-10-421
11. Edgar RC. MUSCLE: a multiple sequence alignment method with reduced time and space complexity. *BMC Bioinformatics* 2004;**5**:113. 10.1186/1471-2105-5-113

12. Ban H, Sato S, Yoshikawa S, et al. Genome analysis of *Parmales*, the sister group of diatoms, reveals the evolutionary specialization of diatoms from phago-mixotrophs to photoautotrophs. *Commun Biol* 2023;**6**:697. 10.1038/s42003-023-05002-x
13. Peers G, Price NM. Copper-containing plastocyanin used for electron transport by an oceanic diatom. *Nature* 2006;**441**:341–344. 10.1038/nature04630
14. Tucker SJ, Fuessel J, Freel KC, et al. A high-resolution diel survey of surface ocean metagenomes, metatranscriptomes, and transfer RNA transcripts. *bioRxiv* 2025. 10.1101/2025.09.15.676277
15. Minh BQ, Schmidt HA, Chernomor O, et al. IQ-TREE 2: New models and efficient methods for phylogenetic inference in the genomic era. *Mol Biol Evol* 2020;**37**:1530–1534. 10.1093/molbev/msaa015
16. Kalyaanamoorthy S, Minh BQ, Wong TKF, et al. ModelFinder: fast model selection for accurate phylogenetic estimates. *Nat Methods* 2017;**14**:587–589. 10.1038/nmeth.4285
17. Mirdita M, Schütze K, Moriwaki Y, et al. ColabFold: making protein folding accessible to all. *Nat Methods* 2022;**19**:679–682. 10.1038/s41592-022-01488-1
18. Cianfrocco MA, Wong-Barnum M, Youn C, et al. COSMIC2: A scientific gateway for cryo-electron microscopy structure determination. *Practice and Experience in Advanced Research Computing 2017: Sustainability, Success and Impact*. 2017. New York, NY, USA: ACM, 2017. 10.1145/3093338.3093390
19. Berman HM, Westbrook J, Feng Z, et al. The Protein Data Bank. *Nucleic Acids Res* 2000;**28**:235–242. 10.1093/nar/28.1.235
20. Sehnal D, Bittrich S, Deshpande M, et al. Mol* Viewer: modern web app for 3D visualization and analysis of large biomolecular structures. *Nucleic Acids Res* 2021;**49**:W431–W437. 10.1093/nar/gkab314
21. Leyton DL, Rossiter AE, Henderson IR. From self sufficiency to dependence: mechanisms and factors important for autotransporter biogenesis. *Nat Rev Microbiol* 2012;**10**:213–225.

10.1038/nrmicro2733

22. Freel KC, Tucker SJ, Freel EB, et al. New isolate genomes and global marine metagenomes resolve ecologically relevant units of SAR11. *bioRxiv* 2024. 10.1101/2024.12.24.630191
23. Langmead B, Salzberg SL. Fast gapped-read alignment with Bowtie 2. *Nat Methods* 2012;**9**:357–359. 10.1038/nmeth.1923
24. R Core Team. R: A language and environment for statistical computing. 2023. R Foundation for Statistical Computing, Vienna, Austria. https://www.R-project.org/
25. Wickham H. ggplot2: Elegant graphics for data analysis. Springer-Verlag New York. 2016.
26. Delmont TO, Kiefl E, Kilinc O, et al. Single-amino acid variants reveal evolutionary processes that shape the biogeography of a global SAR11 subclade. *Elife* 2019;**8**:e46497. 10.7554/elife.46497
27. Haro-Moreno JM, Rodriguez-Valera F, Rosselli R, et al. Ecogenomics of the SAR11 clade. *Environ Microbiol* 2020;**22**:1748–1763. 10.1111/1462-2920.14896

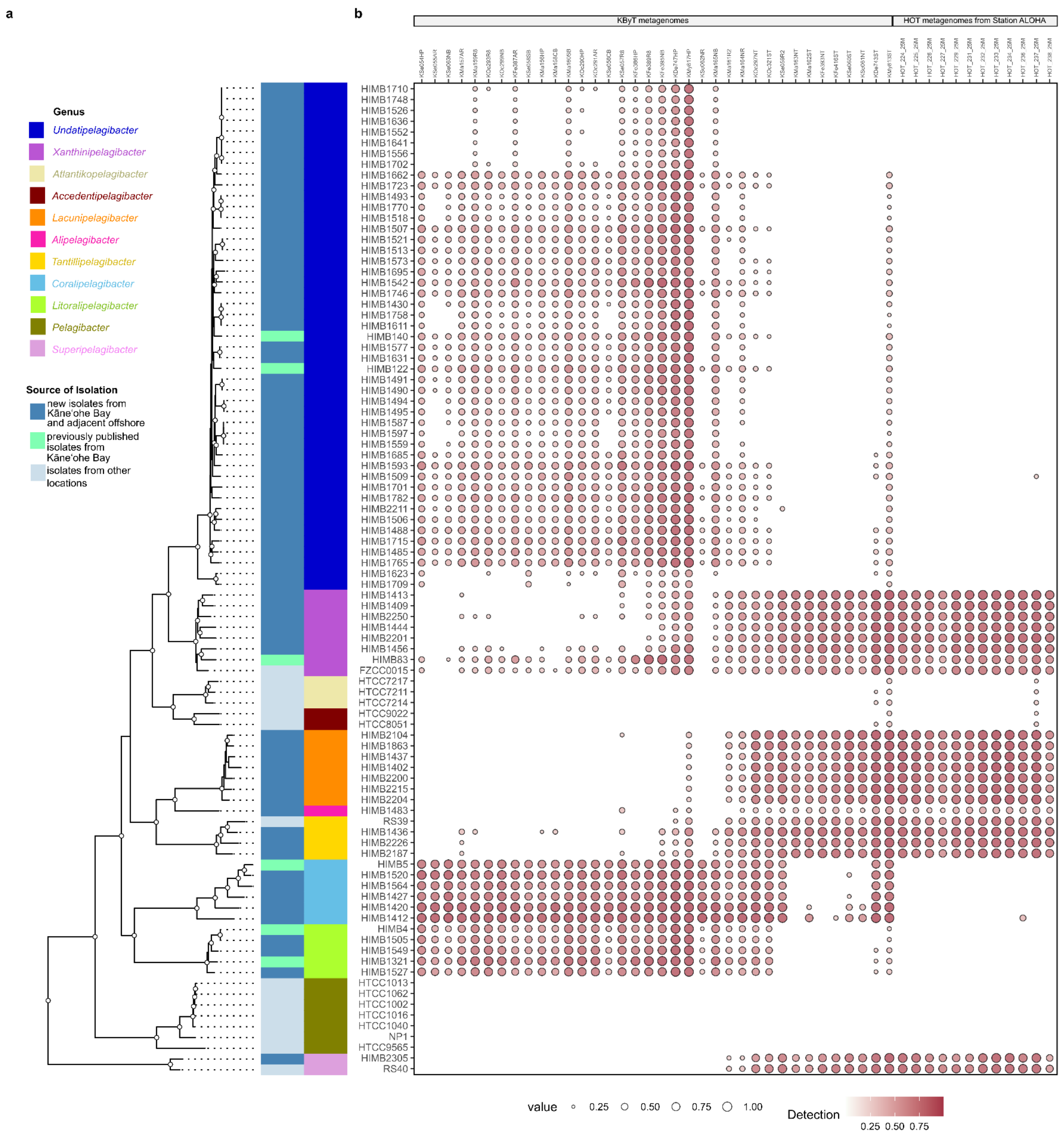

Fig. S1. Phylogenomic position of *Pelagibacteraceae* isolate genomes and their detection in the tropical Pacific. **a)** Phylogenomic tree showing 11 monophyletic clusters within the *Pelagibacteraceae*, their genus assignments, and source of isolation. The majority of *Pelagibacteraceae* strain genomes were isolated from the KByT system. Circles at nodes indicate ultrafast bootstrap support values ≥90% from 1000 replicates. **b)** Detection of *Pelagibacteraceae* genomes in metagenomic samples from KByT and HOT at Station ALOHA. *Pelagibacteraceae* genera that contained KByT isolate genomes were commonly detected within the KByT system and sometimes at Station ALOHA. The three genera not containing KByT isolates were rarely detected in the tropical Pacific, with little detection in samples collected in the KByT system or at Station ALOHA. The order of metagenomes presented follows the order in **Supplementary file 1e**.

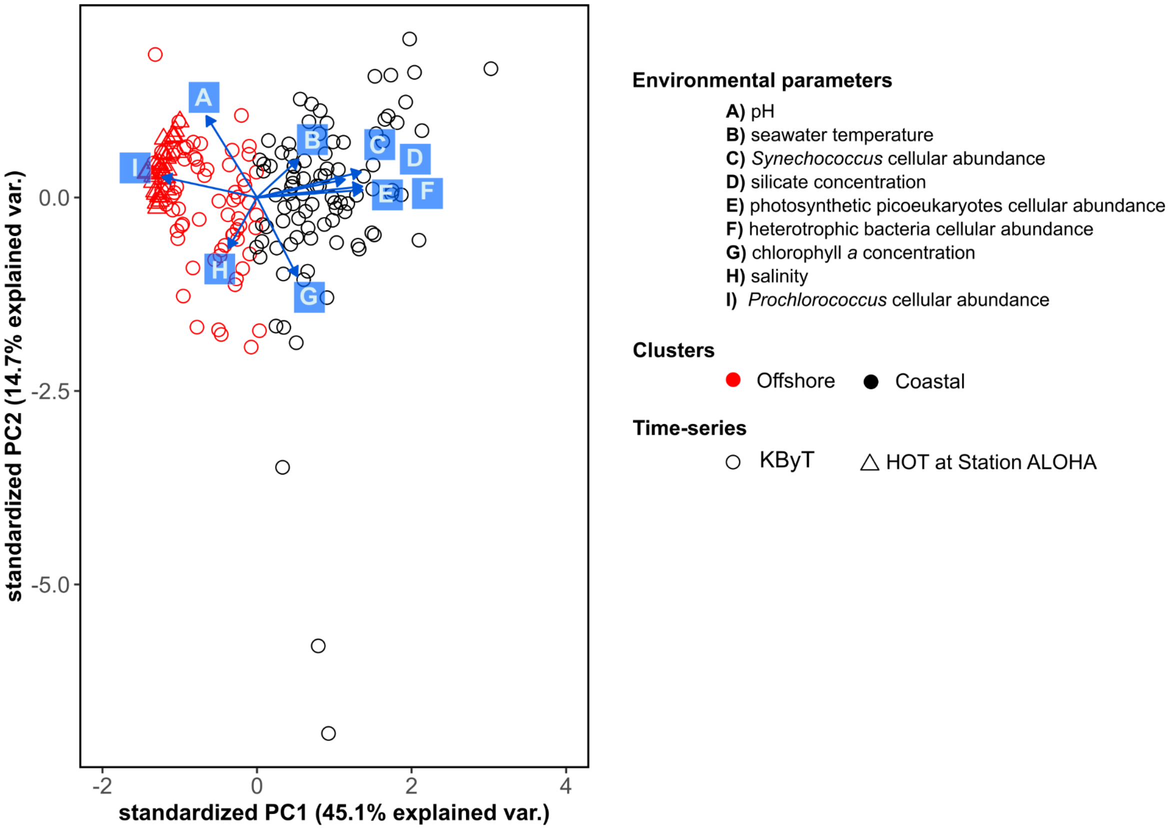

Fig. S2. Surface ocean samples from KByT and HOT at Station ALOHA grouped into two clusters. K-means analyses of environmental parameters from 192 surface ocean samples from KByT and HOT at Station ALOHA grouped into two clusters, herein referred to as coastal and offshore. The underlying environmental covariates from the clustering analysis were mapped using Principal Components Analysis (PCA), which explained 45.1% and 14.7% of the variation across PC1 and PC2, respectively.

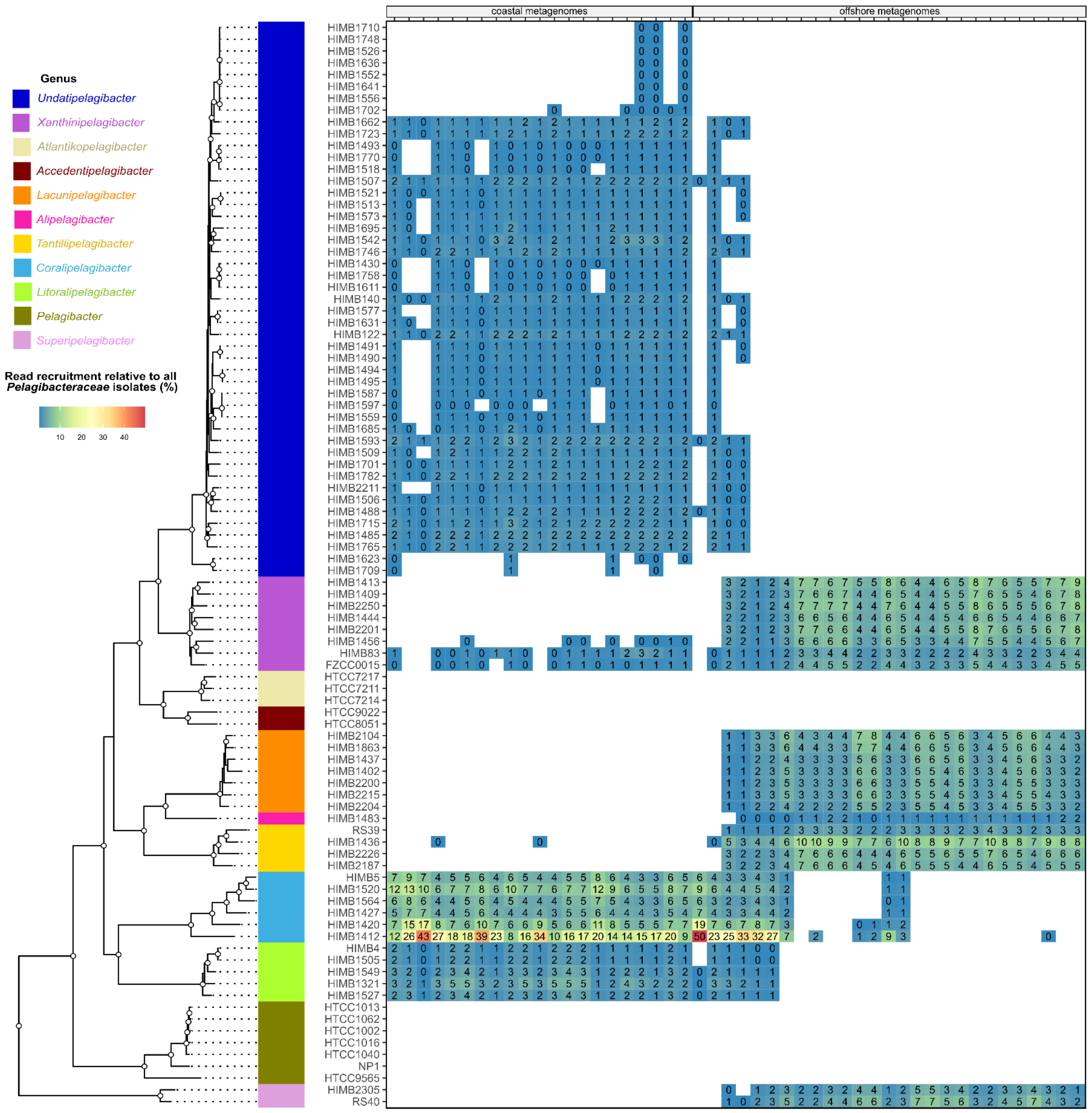

Fig. S3. *Pelagibacteraceae* genomes sharing the same genus show similar biogeographies. Relative read recruitment of each isolate genome across the coastal and offshore environment shows that genomes belonging to the same genus share similar distribution patterns. Genera tend to be either highly prevalent in coastal samples or in the offshore samples, but not both. Data for genomes that are not detected at >0.25 or have a relative recruitment less than 0.25% are not shown. The order of metagenomes presented follows the order in **Supplementary file 1e**.

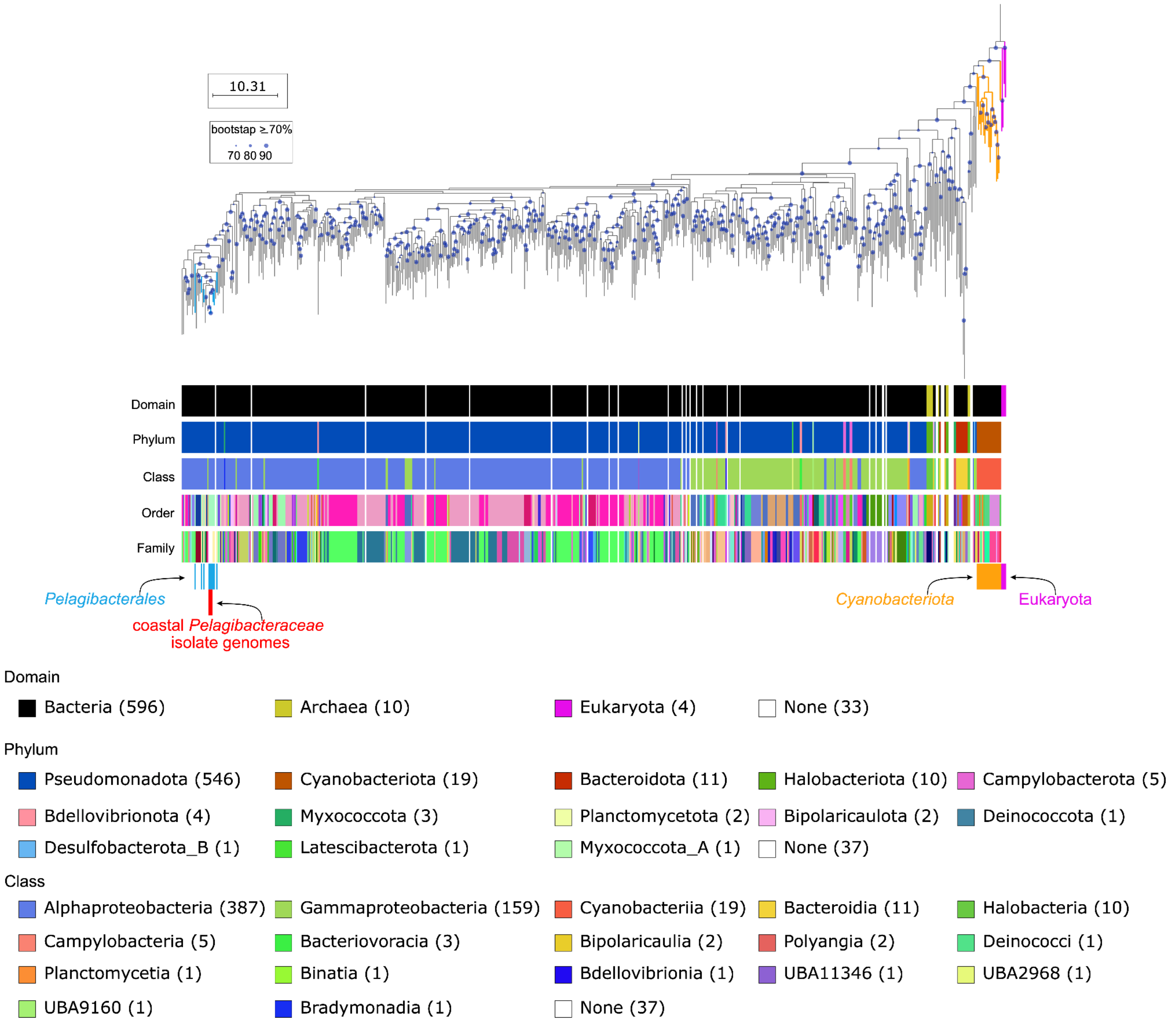

Fig. S4. Phylogeny of *Pelagibacteraceae* plastocyanin-like genes and gene homologs. The taxonomic classifications for each plastocyanin and plastocyanin-like gene are identified by color down to the family-level, with the associated taxonomic assignment specified for domain-, phylum-, and class-levels. Plastocyanin-like gene sequences belonging to the order *Pelagibacterales* are highlighted in aqua. Plastocyanin genes from *Cyanobacteriia* and eukaryotes (highlighted orange and purple, respectively) form distinct clades separate from plastocyanin-like genes in non-photosynthetic (presumably heterotrophic) bacteria. The tree was constructed from aligned plastocyanin-like protein sequences from coastal *Pelagibacteraceae* isolate genomes, protein sequences that were annotated to KEGG Ortholog ID K02638 (plastocyanin; *petE*) in a set of clustered proteins from the GlobDB release 226 and passed a BLAST search using the protein sequences of the *Pelagibacteraceae* plastocyanin-like genes, and four outgroup eukaryotic sequences.

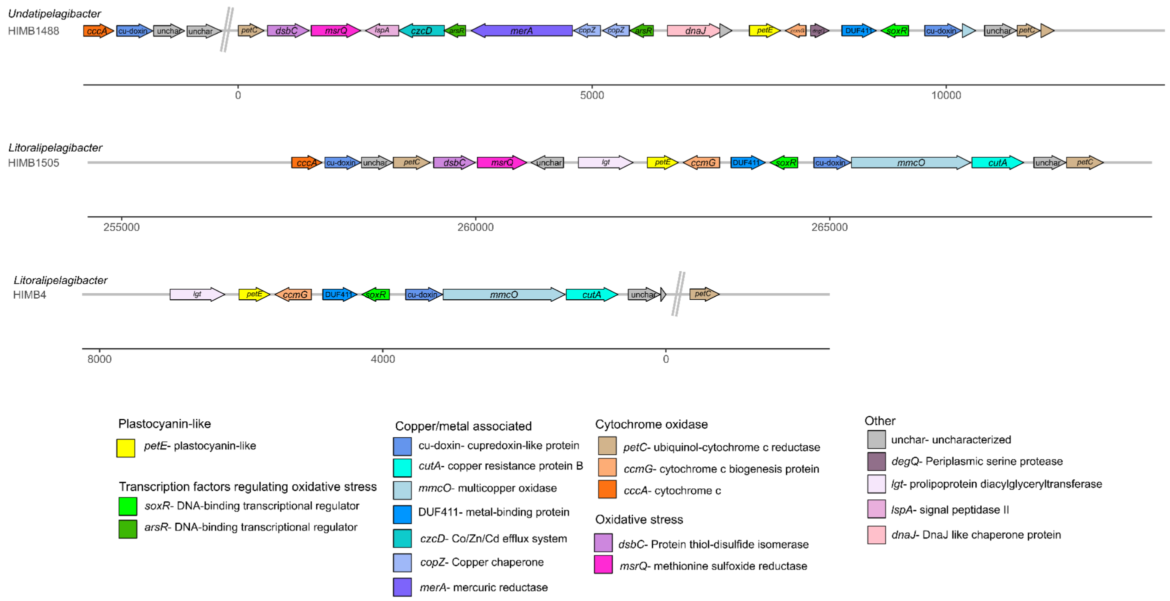

Fig. S5. Genomic regions of plastocyanin-like genes in coastal *Pelagibacteraceae*. Genes surrounding plastocyanin-like genes within *Pelagibacteraceae* genomes are related to cytochrome oxidases, copper/metal enzymes, chaperones, resistance proteins, and efflux systems, and regulating or coping with oxidative stress. These genes are consistent with the hypothesis that the plastocyanin-like gene may be involved in electron transport and contain copper. The region in HIMB4 is reversed. Two slashes mark the end of a contig. Both HIMB4 and HIMB1488 were assembled into a single contig, whereas HIMB1505 is composed of two contigs.

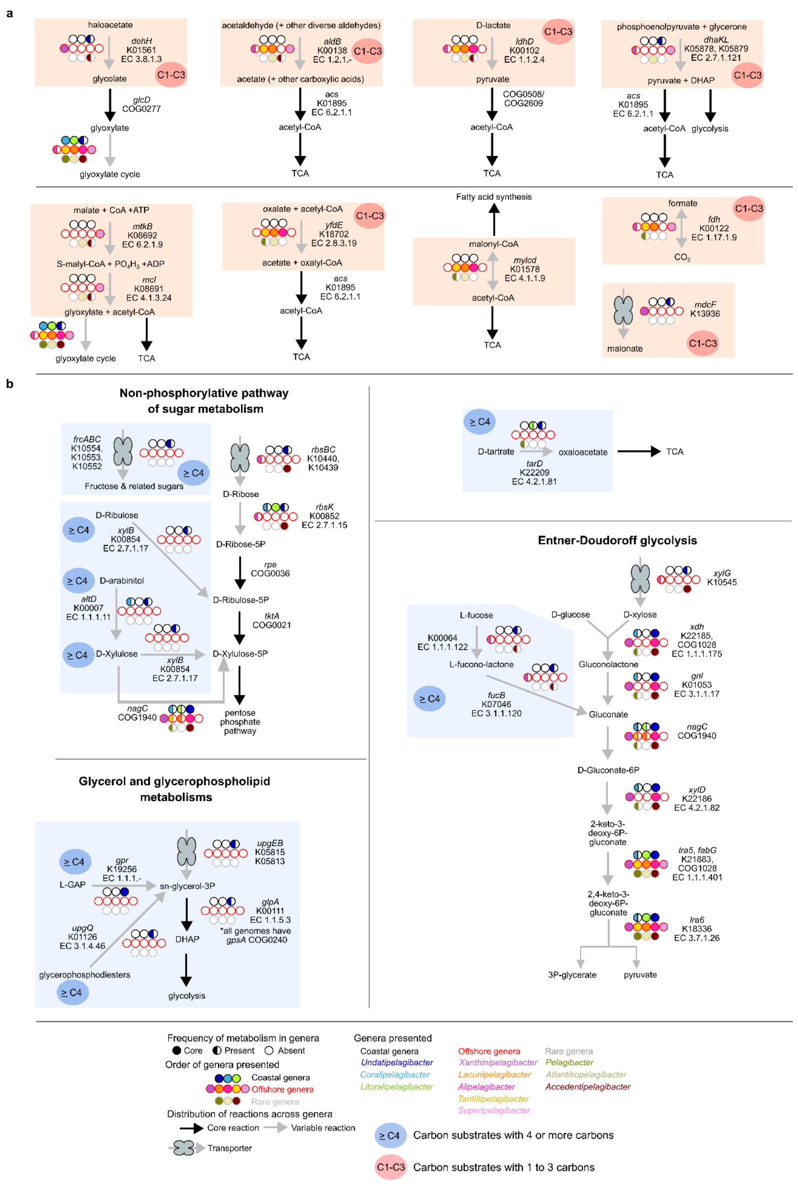

Fig. S6. **Carbon specialization of offshore and coastal *Pelagibacteraceae*. a)** Offshore genera specialize on small C1-C3 carbons that predominantly enter the citric acid cycle (TCA) or the glyoxylate cycle. **b**) Coastal genera, especially *Undatipelagibacter*, specialize on slightly larger sugars (≥C4) that typically enter glycolysis or the pentose phosphate pathway. Blue boxes emphasize the metabolic steps enriched in genomes from coastal genera, whereas orange boxes emphasize the metabolic steps enriched in genomes from offshore genera. The Entner-Doudoroff (ED) glycolytic pathway does not show a coastal or offshore distribution, but was found core only among genomes belonging to *Undatipelagibacter*, *Xanthinipelagibacter*, *Alipelagibacter*, and *Accedentipelagibacter*. L-GAP: L-glyceraldehyde 3-phosphate; TCA: citric acid cycle; CoA: Coenzyme A; ATP: adenosine triphosphate; ADP: adenosine diphosphate; DHAP: dihydroxyacetone phosphate.

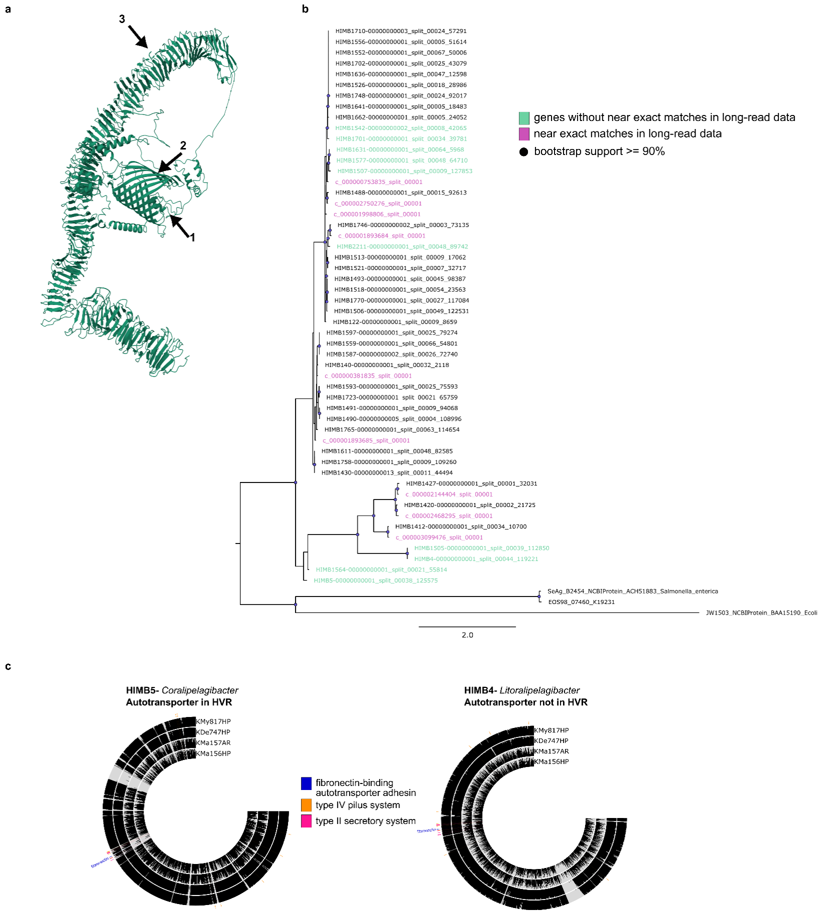

Fig. S7. **Protein structure models and homolog searches with long-read metagenomes support autotransporter adhesion genes in coastal *Pelagibacteraceae***. **a)** A protein structure model of a gene annotated as an autotransporter adhesin from KByT long-read metagenomes that shares close sequence similarity to gene sequences in *Pelagibacteraceae* genomes has characteristics expected of autotransporter proteins: 1) α-helical linker, 2) β-barrel translocator domain, and 3) passenger domains containing β helices (Leyton et al., 2012). **b)** Homolog searches to autotransporter adhesin genes in long-read metagenomes recovered nine non-redundant gene sequences that were near exact matches to most gene sequences in *Pelagibacteraceae* genomes (33 of 43). **c)** Patterns of detection of short-read metagenomic read recruitment data across genes in two genomes of *Pelagibacteraceae* show that autotransporter adhesion genes are sometimes located in hypervariable regions and sometimes not. Genes for each *Pelagibacteraceae* genome are ordered by genome synteny with detection values of read recruitment data per gene shown as a bar per metagenomic sample. Areas of low detection are indicative of hypervariable regions (HVRs), and the autotransporter adhesin genes were located in HVRs within the HIMB5 genome, but not within HIMB4 genome. The autotransport adhesion genes were always positioned next to genes involved in type IV pilus systems and/or type II secretory systems.

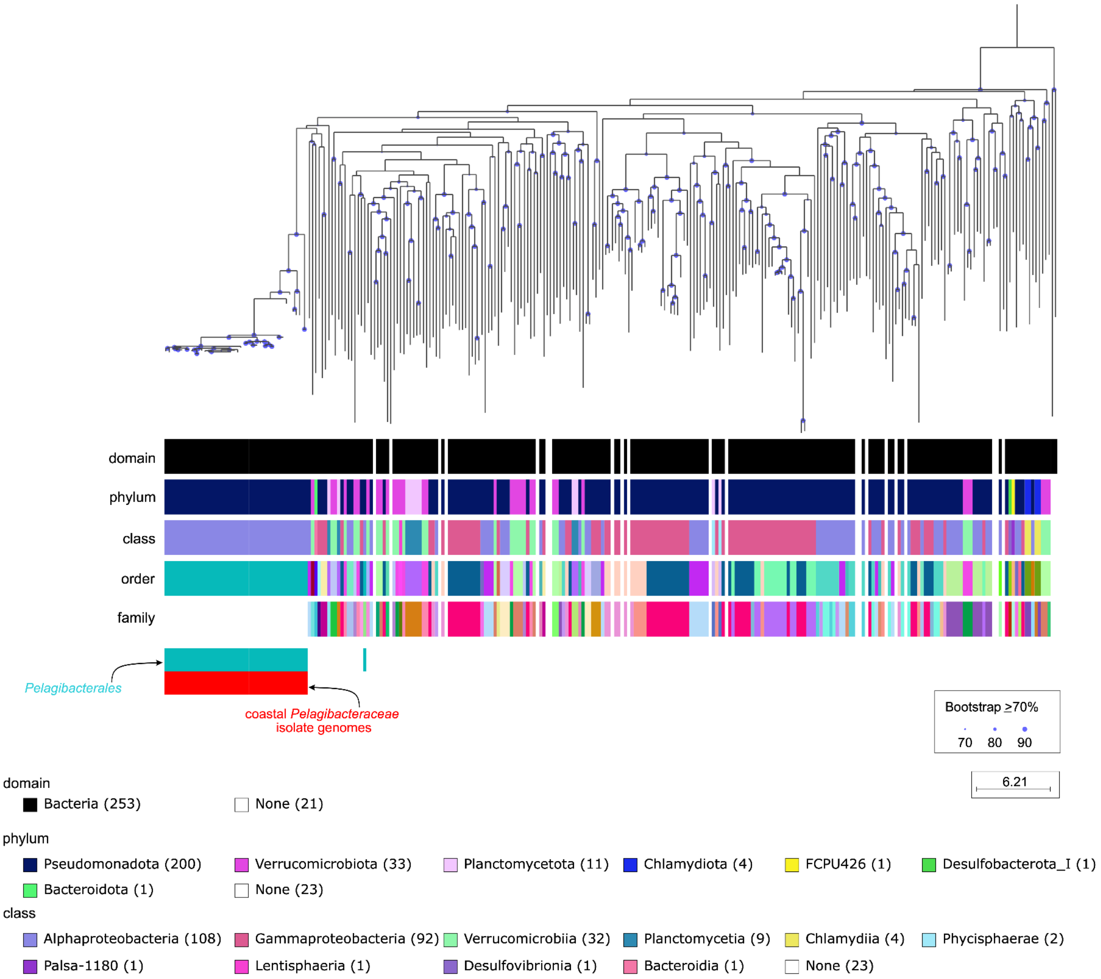

Fig. S8. **Phylogeny of *Pelagibacteraceae* autotransporter adhesion genes and gene homologs**. The taxonomic classification for each autotransport adhesion gene is specified by color categories down to the family-level, with the associated taxonomic assignment specified for domain-, phylum-, and class-levels. All autotransporter adhesion gene sequences belonging to the order *Pelagibacterales* are highlighted in aqua, including gene sequences from coastal *Pelagibacteraceae* isolate genomes and a gene sequence from *Fontibacterium*. The tree was constructed from aligned protein sequences from coastal *Pelagibacteraceae* isolate genomes (in red), protein sequences that were annotated to KEGG ortholog K19231 (fibronectin-binding autotransporter adhesin) in the set of clustered proteins from the GlobDB release 226 and passed a blast search using the protein sequences of the *Pelagibacteraceae* autotransporter adhesion genes, and two outgroup sequences.

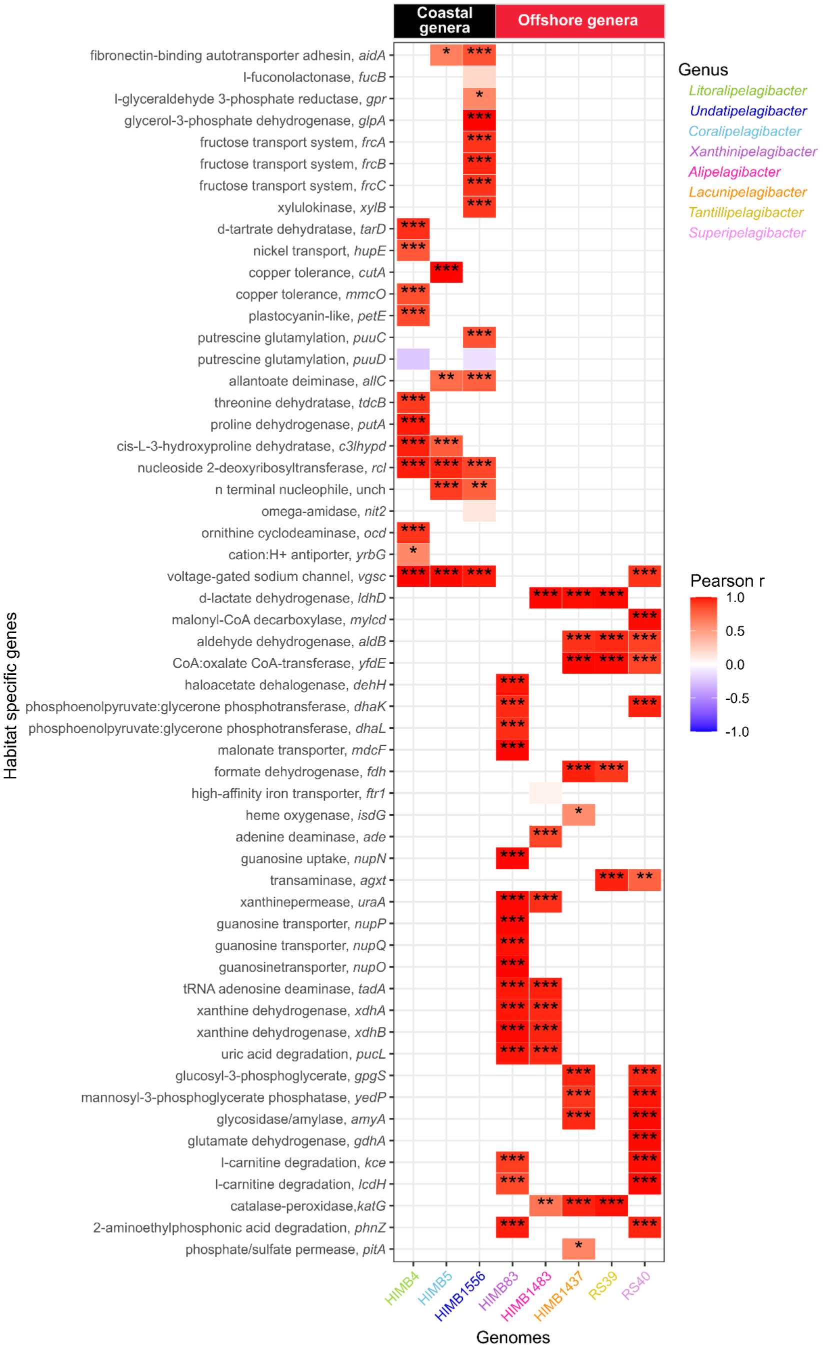

Fig. S9. **Coverages of habitat specific genes correlate with coverage of *Pelagibacteraceae* genomes globally**. Pearsons correlations between the coverage of habitat specific genes and the coverage of *Pelagibacteraceae* genomes to which these genes belong to were mostly positive and statistically significant. Genomes are colored by their genus assignment and labeled for their coastal or offshore distribution within KByT. Gene coverage and genome coverage were transformed with log+1 prior to correlation analysis.

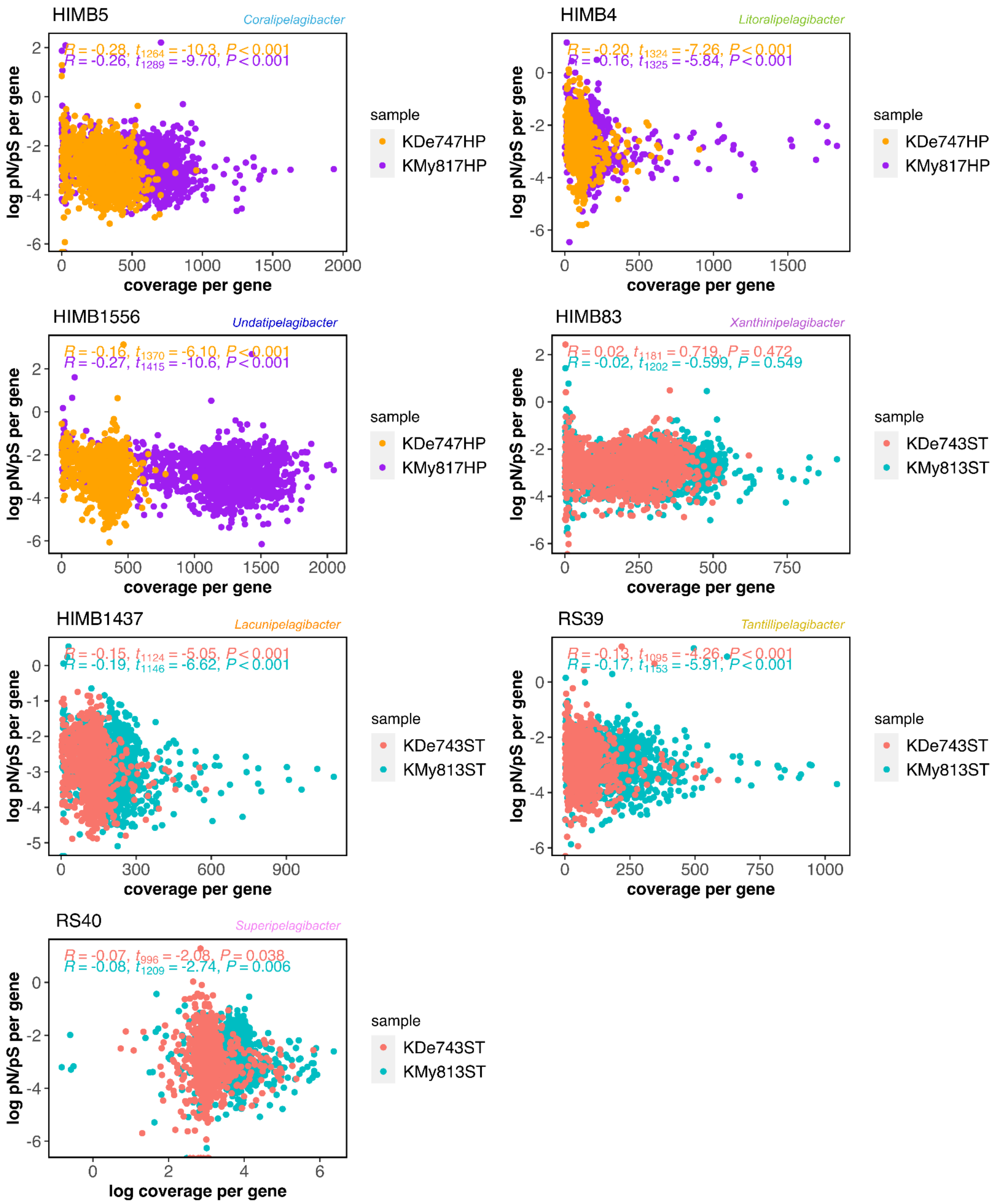

Fig. S10. **Relationship between pN/pS values and gene coverages in *Pelagibacteraceae* genomes**. Reads from deeply sequenced metagenomes collected at coastal (KDe747HP, KMy817HP) and offshore (KDe743ST, KMy813ST) Kāneʻohe Bay Time-series (KByT) stations were recruited to genomes for each of the seven *Pelagibacteraceae* genera abundant in the KByT system. The relationship between coverage per gene and pN/pS values per gene were compared for each of the genomes, showing that gene coverage did not correlate with pN/pS value. Data were normalized with log transformations. pN/pS: proportion of non-synonymous to synonymous.

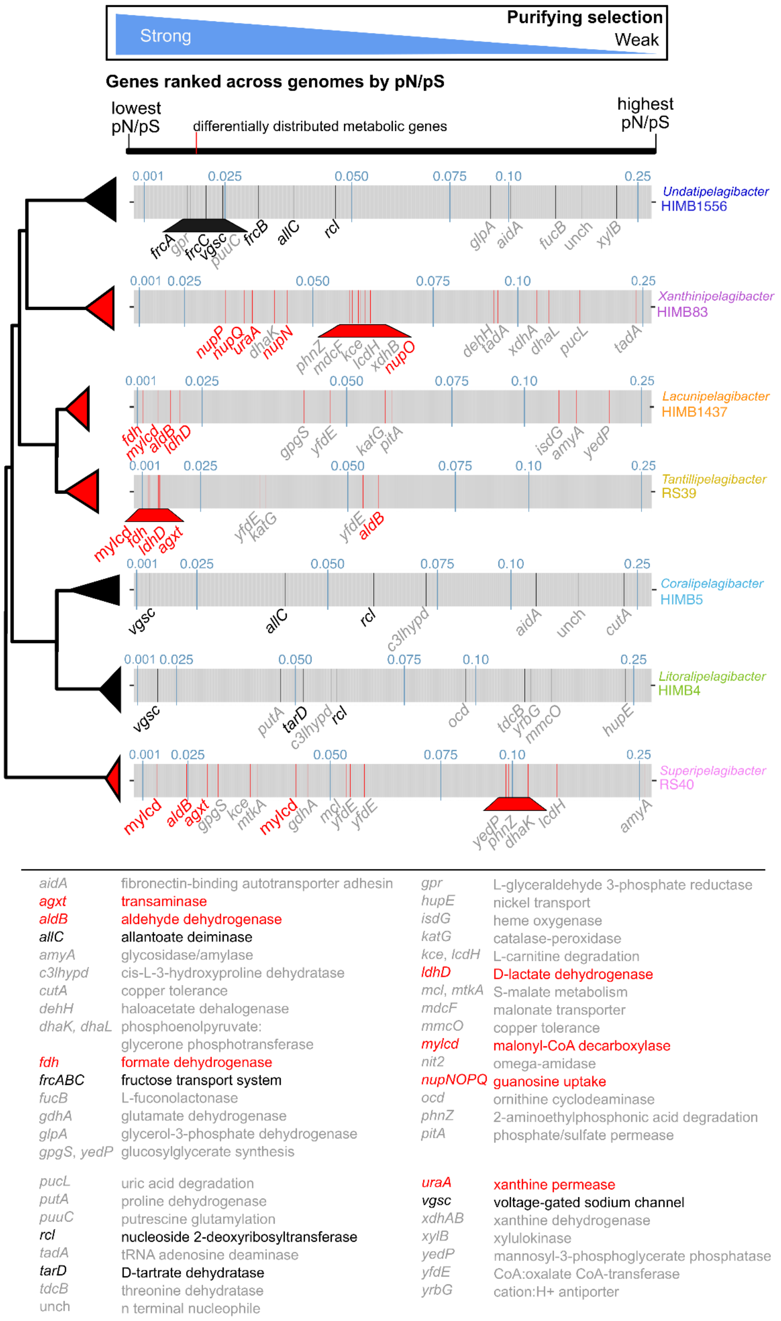

Fig. S11. **Selective pressures on habitat-specific genes in *Pelagibacteraceae***. All genes in a genome are ordered by lowest pN/pS value (high purifying selection) to highest pN/pS values (low purifying selection). Genes that are differentially distributed between offshore and coastal *Pelagibacteraceae* genera in **Fig. 4** are colored red (offshore) or black (coastal) across the genomes. Gene labels that are colored in red or black are those that were highlighted in the main text and shown in **Fig. 5**. pN/pS values of 0.01, 0.025, 0.05, 0.075, 0.1, and 0.25 are shown in blue. Genomes are ordered by the phylogenomic relationships. pN/pS: proportion of non-synonymous to synonymous.

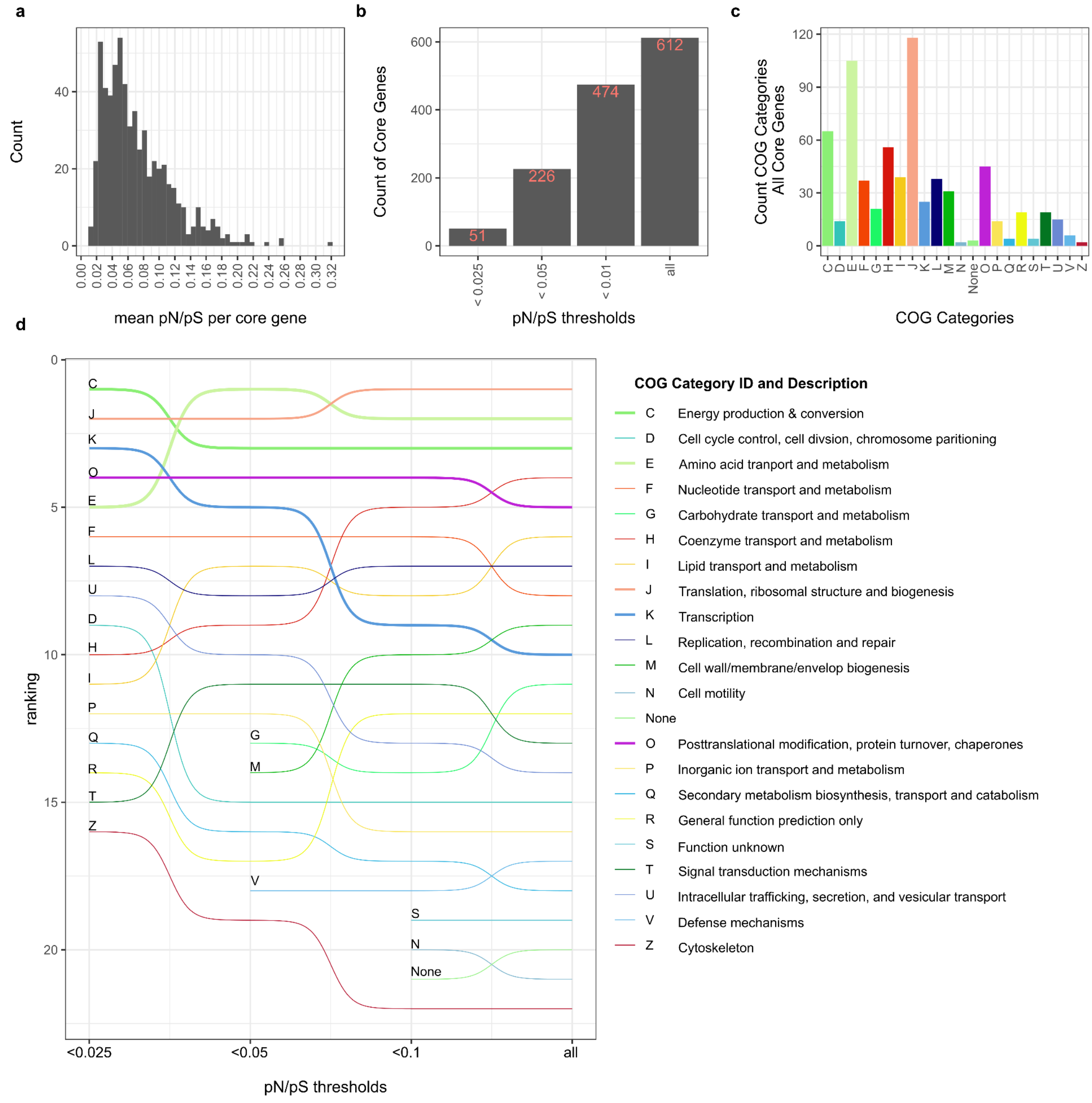

Fig. S12. **pN/pS distributions and functional category composition within *Pelagibacteraceae* core genes**. **a**) Low pN/pS values are typical of *Pelagibacteraceae* core. **b**) The number of genes found at four different pN/pS thresholds: <0.025, <0.05, <0.01, and all genes. **c**) The number of core genes found in each of the COG categories. COG category descriptions are found in panel d. **d**) At each of the different pN/pS thresholds, COG categories were ranked by their count. There was consistency in top ranked COG categories across the two lowest pN/pS thresholds.

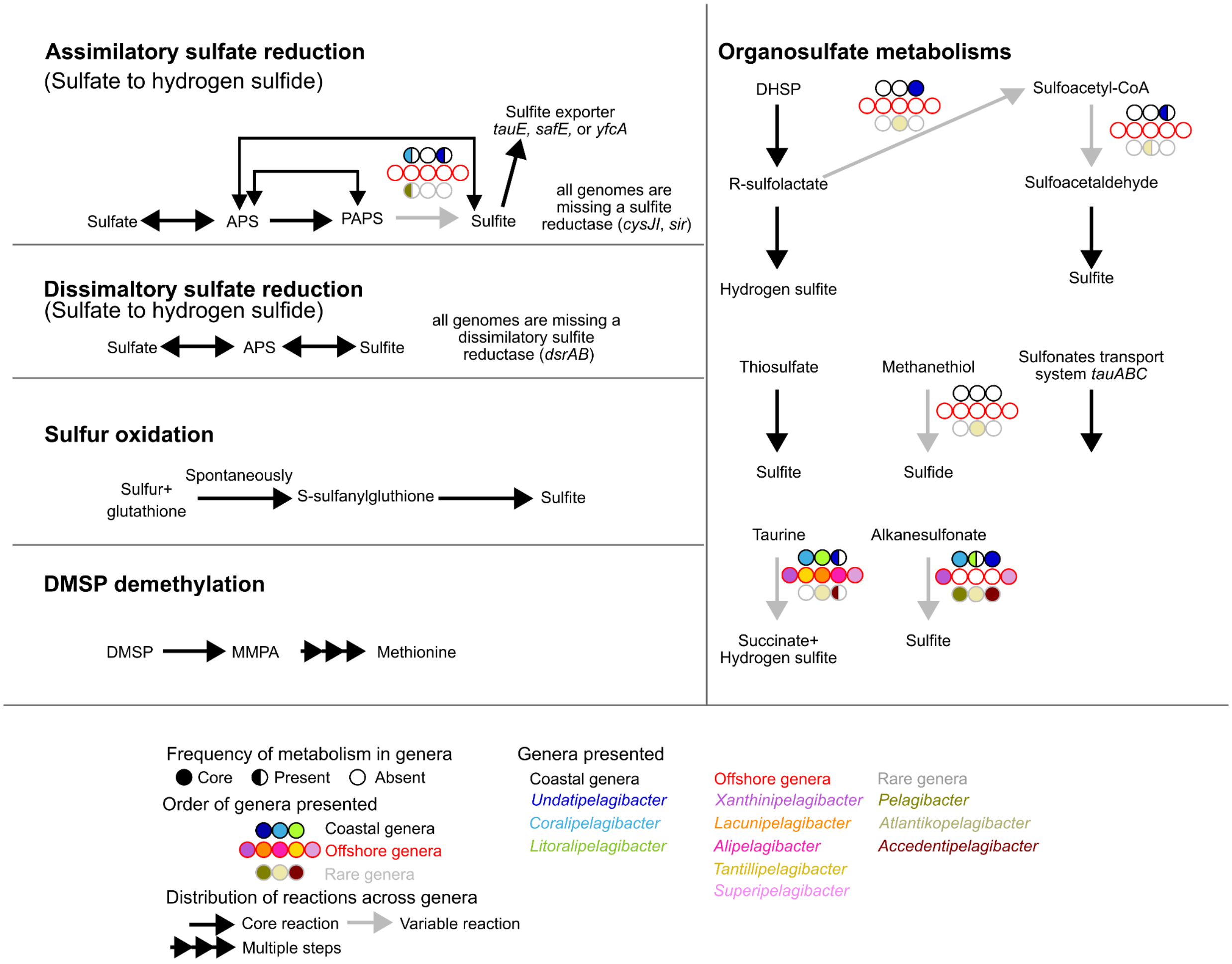

Fig. S13. **Sulfur metabolisms within *Pelagibacteraceae***. *Pelagibacteraceae* genomes have incomplete dissimilatory and assimilatory sulfur reduction pathways and the capacity to utilize a wide variety of organosulfate compounds. APS: adenylyl sulfate, PAPS: 3'-phosphoadenylyl sulfate, DHSP: 2,3-dihydroxypropane-1-sulfonate, DMSP: dimethylsulfoniopropionate, MMPA: methylmercaptopropionate.

Legends Supplementary files 1-3. (separate files)

Supplementary file 1a. *Pelagibacteraceae* isolate genomes used in this reference, isolation source, and genome characteristics. Accessions refer to RefSeq or NCBI databases unless an asterik is present, which denotes the accession ID is within the JGI-IMG database.

Supplementary file 1b. Summary of *Pelagibacteraceae* genome-wide average identity (gANI).

Supplementary file 1c. Environmental parameters for surface seawater samples from the Kāne'ohe Bay Time-series (KByT) and the Hawaii Ocean Time Series (HOT) at Station ALOHA. Metagenome accession IDs are also provided. ^ Indicates that only phosphate and nitrate+nitrite concentrations from KByT samples are reported because higher accuracy low-level estimates of nitrate+nitrite and phosphate concentrations were available for Station ALOHA.

Supplementary file 1d. Differences in biogeochemical parameters across coastal and offshore kmeans clusters for surface seawater samples from the Kāne'ohe Bay Time-series (KByT) and the Hawaii Ocean Time Series (HOT) at Station ALOHA. Comparisons of biogeochemical parameters between kmeans clusters use a one-way ANOVA. Significance (uncorrected P values) and F values are shown. ^ Indicates that only phosphate and nitrate+nitrite concentrations from offshore KByT samples were used in these summary statistics because higher accuracy low-level estimates of nitrate+nitrite and phosphate concentrations were available for Station ALOHA.

Supplementary file 1e. Relative abundance (%) of *Pelagibacteraceae* genera across metagenomic samples.

Supplementary file 2a. Pangenomic analyses of 92 *Pelagibacteraceae* isolate genomes. gANI: genome-wide average nucleotide identity, GC: gene cluster.

Supplementary file 2b. Categories of gene clusters based on distribution in genera and their functional annotations.

Supplementary file 2c. Categories of functional clusters based on distribution in genera and their annotations.

Supplementary file 2d. Distribution of genes associated with molybdenum enzyme utilization and molybdenum cofactor biosynthesis displayed as the number of genomes with that gene per genus relative to the total number of genomes per genus. NA denotes that the gene was not found in any *Pelagibacteraceae* genomes.

Supplementary file 2e. Distribution of genes associated with purine salvage and catabolism displayed as the number of genomes with that gene per genus relative to the total number of genomes per genus. NA denotes that the gene was not found in any *Pelagibacteraceae* genomes.

Supplementary file 2f. Distribution of habitat-specific genes involved in nutrient acquisition and utilization and osmotic and oxidative stress displayed as the number of genomes with that gene per genus relative to the total number of genomes per genus. NA denotes that the gene was not found in any *Pelagibacteraceae* genomes.

Supplementary file 2g. Summary table of habitat-specific metabolisms and their relative frequency in genomes with offshore and coastal distributions, as well as their relative frequency across individual genera.

Supplementary file 2h. Distribution of genes involved in carbon metabolism, amino acid, vitamin, and cofactor synthesis, methionine salvage, nutrient acquisition and utilization, and adhesion displayed as the number of genomes with that gene per genus relative to the total number of genomes per genus. NA denotes that the gene was not found in any *Pelagibacteraceae* genomes.

Supplementary file 2i. Global metagenomes used to assess the presence of potential habitat specificity genes in regions beyond KByT and the relative abundance of select *Pelagibacteraceae* genomes in these metagenomes.

Supplementary file 3a. Log pN/pS across genes with shared KOfam assignments within the genus Coralipelagibacter (n=6) varies more between genes in a given sample than between samples for a given gene or for genomes within a genus.

Supplementary file 3b. Non-outlier gene coverage summary for read recruitment of deeply sequenced metagenomes from coastal Kāneʻohe Bay and the adjacent offshore to genome representatives used in pN/pS analyses.

Supplementary file 3c. Summary of pN/pS values per gene across genomes.

Supplementary file 3d. Log pN/pS values across genes varies more between genes in a given sample than between samples for a given gene. The pound sign (#) indicates log transformation for one of the factors.

Supplementary file 3e. Core *Pelagibacteraceae* genes and their pN/pS ratios in the KByT system. Mean pN/pS values for core *Pelagibacteraceae* genes with shared KOfam annotations examined from genomes representing the seven *Pelagibacteraceae* genera that are highly abundant in the tropical Pacific. Genes are ordered from lowest mean pN/pS to highest mean pN/pS. COG categories associated with each gene call are also included. pN/pS: proportion of non-synonymous to synonymous.
